# Glutamic Acid–Chelated Cobalt Stabilizes G-Quadruplexes and Selectively Suppresses Hepatocellular Carcinoma Growth

**DOI:** 10.32604/or.2026.074144

**Published:** 2026-03-23

**Authors:** Kuan-Hao Lin, Yu-Ju Lin, Yu-Bin Hong, Meng-Huai Hsu, Zhen-Xiang Liao, Shuo-Yu Chang, Chiou-Hwa Yuh

**Affiliations:** 1 Institute of Molecular and Genomic Medicine, National Health Research Institutes, Zhunan, Miaoli, Taiwan; 2 Institute of Bioinformatics and Structural Biology, National Tsing Hua University, Hsinchu, Taiwan; 3 Department of Biological Science and Technology, National Yang Ming Chiao Tung University, Hsinchu, Taiwan; 4 Program in Environmental and Occupational Medicine, Kaohsiung Medical University, Kaohsiung, Taiwan

**Keywords:** Liver cancer, glutamic acid cobalt chelate, *tert* transgenic zebrafish, G-quadruplex stabilization, phenotypic response surface, *KRAS* promoter, zebrafish xenograft

## Abstract

**Objectives:**

Hepatocellular carcinoma (HCC) has limited systemic options with substantial toxicity. G-quadruplex (G4) structures in oncogene promoters are attractive but challenging drug targets. This study aimed to determine whether glutamic acid–chelated cobalt (GACC) is a G4-active scaffold with anti-HCC efficacy and favorable *in vivo* safety, and whether an AI-guided phenotypic response surface (PRS) can optimize less toxic combinations.

**Methods:**

Anticancer activity was tested in HCC cell lines (PLC/PRF/5, Hep3B, HepG2) and non-transformed THLE-2 hepatocytes (CCK-8, IC_50_). *In vivo* safety/efficacy were assessed in zebrafish embryo toxicity assays, a Hep3B xenograft model, and a tert-overexpressing transgenic zebrafish model, with hepatotoxicity monitored in a liver-fluorescent reporter line. Target engagement was examined by docking, native PAGE, a KRAS promoter G4 DNA polymerase stop assay, BG4 immunofluorescence, and KRAS qPCR. PRS was used to optimize GACC–metformin–regorafenib combinations.

**Results:**

GACC reduced HCC viability (IC_50_ ~86–115 µM) and showed low embryotoxicity (IC_50_ 6.87 mM). In zebrafish xenografts, GACC (50 µM) reduced Hep3B tumor fluorescence by ~90% without detectable hepatotoxicity, whereas sorafenib decreased liver size/fluorescence. In tert-overexpressing zebrafish, GACC suppressed proliferation and Wnt/β-catenin–associated transcripts and reduced mitotic figures and nuclear atypia. Mechanistically, GACC increased KRAS promoter polymerase stalling, enhanced nuclear G4 signal, and reduced KRAS transcripts. PRS identified an off-grid triple combination that reduced PLC/PRF/5 viability to 19% while maintaining THLE-2 viability at 52% and preserving zebrafish development.

**Conclusion:**

GACC is a G4-active cobalt–glutamate scaffold with anti-HCC activity and favorable zebrafish safety, and a zebrafish-plus-PRS workflow enables rational, less toxic combination design.

## 1 Introduction

Cancer remains one of the leading causes of morbidity and mortality worldwide, accounting for nearly 10 million deaths each year. Despite significant therapeutic advances, cancer continues to pose a major global health challenge because of its rising incidence, biological complexity, and the lack of effective treatments for several tumor types [1].

Hepatocellular carcinoma (HCC) is the most common primary liver malignancy and remains a major cause of cancer mortality worldwide. Based on the GLOBOCAN 2022 estimates, liver cancer accounts for ~0.87 million new cases and ~0.76 million deaths globally, reflecting its high fatality burden [2]. Major etiological drivers include chronic hepatitis B and C virus infections, aflatoxin exposure, alcohol-associated liver disease, and the rapidly growing contribution of metabolic dysfunction-associated steatotic liver disease (MASLD; previously termed NAFLD/MAFLD) [3–5]. Although surveillance programs have improved early detection, many patients are still diagnosed at intermediate or advanced stages, when they are no longer candidates for curative resection or liver transplantation [6].

Conventional cytotoxic chemotherapy has historically played only a minor role in HCC management. HCC often exhibits intrinsic chemoresistance, and regimens based on doxorubicin, cisplatin, and 5-fluorouracil generally yield low objective response rates, limited survival benefits, and considerable systemic toxicity, particularly in patients with cirrhosis or impaired liver function [7–9]. Recent reviews highlight that the modest efficacy and poor tolerability of cytotoxic chemotherapy have led to its progressive abandonment and a shift toward targeted therapies and, more recently, immunotherapy-based combinations as the main systemic options for advanced HCC [8–10]. However, the overall therapeutic landscape remains far from optimal.

Systemic therapies are the cornerstone of treatment for advanced HCC. Sorafenib, a multikinase inhibitor, was the first agent to demonstrate an overall survival benefit, although the benefit is modest, extending life by only 2–3 months in most patients [11]. Subsequent agents such as lenvatinib, regorafenib, and the combination of atezolizumab and bevacizumab have improved outcomes in selected populations but are associated with variable efficacy and toxicity profiles [12,13]. Resistance frequently emerges, and adverse effects, including hepatotoxicity, limit long-term use [14]. There is thus a critical need for novel agents and combinations that can effectively target tumor cells while sparing normal hepatocytes.

Recent advances in cancer biology have identified DNA G-quadruplex (G4) structures as promising therapeutic targets. G4s are non-canonical secondary structures formed by guanine-rich sequences and stabilized by Hoogsteen hydrogen bonding and monovalent cations. They are enriched in the promoter regions of oncogenes such as *KRAS*, *MYC*, and *BCL-2* and can regulate gene expression by modulating transcription and DNA replication dynamics [15–17]. Stabilization of G4s in cancer cells has been shown to downregulate oncogene expression and inhibit tumor growth [18,19].

Several G4-stabilizing ligands have been developed, including TMPyP4, BRACO-19, and CX-3543, which show preclinical efficacy by suppressing transcription, inducing G2/M cell-cycle arrest, and promoting apoptosis [20–22]. TMPyP4 stabilizes G4 and is able to suppress further c-MYC transcriptional activation [23]. However, their clinical translation has been limited by poor selectivity, off-target to duplex DNA, and systemic toxicity. For example, TMPyP4, although a potent G4 binder, also intercalates into duplex DNA and perturbs cellular redox balance [24], whereas the clinical development of CX-5461 was halted because of hematologic toxicity [25]. These limitations underscore the need for new G4 ligands with improved tumor specificity and tolerability.

Glutamic acid-chelated cobalt (GACC) is a metal-organic complex initially designed for agricultural use. Its molecular structure, built on a glutamic acid backbone coordinated with cobalt(II), provides a water-soluble, redox-active scaffold with potential for interactions with nucleic acid structures. Unlike many classical polyaromatic G4 ligands, GACC has favorable solubility and formulation properties. Our studies suggested that GACC stabilizes G4 structures in *KRAS* oncogene promoters, downregulates *KRAS* expression in hepatoma models, exhibits low cytotoxicity in normal hepatocytes, and is well tolerated in zebrafish embryos, supporting a favorable safety profile.

Zebrafish (*Danio rerio*) have emerged as a powerful platform for cancer modeling and drug discovery because of their genetic homology to humans, optical transparency during early development, and suitability for medium- to high-throughput *in vivo* assays [26,27]. Transgenic zebrafish expressing liver-specific oncogenes, such as *HBx*, *src*, and *tert*, recapitulate key histological and molecular features of human HCC [28,29]. Fluorescently tagged lines enable real-time visualization of hepatotoxicity and tumor burden, providing an efficient means of evaluating both efficacy and safety *in vivo* [30].

This study aimed to characterize the structural, pharmacological, and biological properties of GACC in the context of HCC therapy. We evaluated GACC’s ability to stabilize G4 DNA, suppress oncogene transcription, and inhibit tumor growth using human hepatoma cell lines and zebrafish models to assess systemic toxicity and *in vivo* efficacy. We further applied a phenotypic response surface (PRS) platform to explore rational combinations of GACC with established agents, such as metformin and regorafenib. Exploratory antiviral and antimicrobial assays were also performed and are reported as preliminary observations. Together, these studies seek to position GACC as a novel G4-active scaffold and to illustrate a zebrafish- and PRS-guided framework for designing combination strategies in HCC.

## 2 Materials and Methods

Key resources and RRIDs are listed in Supplementary Table S1; RRIDs are also provided at first mention in the text where available.

### 2.1 Cell Lines and Culture

Human hepatoma cell lines PLC/PRF/5 (BCRC 60223, Hsinchu City, Taiwan), Hep3B2.1-7 (Hep3B; BCRC 60434), and HepG2 (BCRC 60025) were purchased from the Bioresource Collection and Research Center (BCRC), Food Industry Research and Development Institute (Hsinchu, Taiwan). The immortalized hepatocyte line THLE-2 (CRL-2706) was obtained from American Type Culture Collection (ATCC, Manassas, VA, USA). Cells were cultured in Dulbecco’s modified Eagle medium (DMEM, Gibco (Thermo Fisher Scientific), 11965-092, Waltham, MA, USA) supplemented with 10% fetal bovine serum (FBS, Gibco, 16000-044) and 1% penicillin–streptomycin (Gibco, 15140-122) at 37°C in humidified incubator with 5% CO_2_. All cell lines were authenticated by short tandem repeat (STR) profiling within the past 12 months and were confirmed to be mycoplasma-negative before experiments.

### 2.2 Synthesis and Characterization of GACC

Glutamic acid–chelated cobalt (GACC; empirical formula C_5_H_11_CoNO_6_; MW 240.08 g/mol) was provided by Amelio Biomedical Co., Ltd. (New Taipei City, Taiwan). Metformin hydrochloride (MedChemExpress, HY-17471A/CS-1851; CAS 1115-70-4; MedChemExpress, Monmouth Junction, NJ, USA), regorafenib (TargetMol, T1792; CAS 755037-03-7, TargetMol, Boston, MA, USA), and pyridostatin (RR82) trifluoroacetate salt (PDS, Selleck Chemicals, S7444; Houston, TX, USA) were dissolved in dimethyl sulfoxide (DMSO, Sigma-Aldrich, D4540; St. Louis, MO, USA) to prepare stock solutions and diluted into assay media immediately before use. The final DMSO concentration did not exceed 0.1% (v/v). All reagents were analytical grade.

Single-crystal X-ray diffraction (SC-XRD) data were collected on a Bruker D8 Venture diffractometer (Bruker AXS, Karlsruhe, Germany) equipped with a Mo microfocus X-ray source (Kα = 0.71073 Å) and a PHOTON II CMOS detector, with the crystal maintained at 200 K using an Oxford Cryosystems 800+ nitrogen flow device (Oxford Cryosystems Ltd., Oxford, UK). Data collection was performed using APEX3 software (Bruker AXS, version 2021). Data integration and cell refinement were carried out with SAINT (Bruker AXS, version 8.40B), and absorption corrections were applied using SADABS (Bruker AXS, version 2016/2). The structure was solved by SHELX (Bruker AXS, 2018/2) and refined by full-matrix least-squares refinement on F^2^ using SHELXL (2019/1), as implemented in the SHELXTL software package (Bruker AXS). Additional crystallographic and refinement parameters are provided in Supplementary Tables S2–S6. The crystallographic data have been deposited with the Cambridge Crystallographic Data Centre (CCDC). The deposition number is 2521452.

### 2.3 Cell Viability and Determination of IC_**50**_

Cell viability was quantified using the Cell Counting Kit-8 (CCK-8; CK04, Dojindo Molecular Technologies, Kumamoto, Japan). All experiments were performed in at least three independent biological experiments (n ≥ 3), each with three technical replicates per condition. Cells were seeded in 96-well plates (3000 cells/well) and allowed to attach overnight. Cells were treated with GACC over a concentration range of 0–10 mM (vehicle-matched controls) for 72 h. After treatment, 10 µL of CCK-8 reagent was added to each well, and plates were incubated for 2 h at 37°C. Absorbance was measured at 450 nm using a multimode microplate reader (Infinite^®^ M200 PRO, Tecan Group Ltd., Männedorf, Switzerland). Technical replicates were averaged to yield a single value per biological replicate. Half-maximal inhibitory concentration (IC_50_) values and a selectivity index (IC_50__normal/IC_50__HCC) were estimated by nonlinear regression using a four-parameter logistic model (log[inhibitor] vs. response, variable slope) in GraphPad Prism (v10; GraphPad Software, San Diego, CA, USA). Goodness-of-fit was assessed by inspection of residuals and R^2^; curves were accepted only when the fit converged and residuals were randomly distributed.

### 2.4 Zebrafish Husbandry and In Vivo Assays

#### 2.4.1 Husbandry and Ethics

Zebrafish (*Danio rerio*) were housed and maintained at the Taiwan Zebrafish Technology and Resource Center in a recirculating aquatic system at a constant temperature of 28.5°C under a 14:10 h light:dark cycle. Water quality was routinely monitored and maintained within facility target ranges: pH 7.0–7.5 (acceptable 6.5–8.0 with emphasis on stability), conductivity typically 300–1500 µS/cm (recirculating systems commonly 1000–1600 µS/cm), dissolved oxygen >6 mg/L, and ammonia/nitrite near 0 ppm. Fish were fed a standard facility diet. Larvae were first fed beginning at ~5 dpf with live foods (e.g., rotifers/Paramecium) and/or powdered diets 3–4 times daily, then transitioned to larger foods during juvenile stages; adults were fed 2–3 times daily with amounts consumed within ~5 min to minimize overfeeding.

All animal procedures were approved by the Institutional Animal Care and Use Committee of the National Health Research Institutes (NHRI-IACUC-113155-A, Zhunan, Miaoli County, Taiwan) and are reported in accordance with the ARRIVE guidelines. The transgenic line *Tg(fabp10a:EGFP-mCherry)* was used for larval liver fluorescence–based hepatotoxicity readouts, and *Tg(fabp10a:tert)* was used for liver tumor progression studies. The *Tg(fabp10a:EGFP-mCherry)* transgenic zebrafish line was previously generated and characterized by our group [30]. The line expresses EGFP–mCherry under the control of the liver-specific *fabp10a* promoter on a wild-type AB genetic background and enables visualization and quantification of liver morphology and fluorescence intensity *in vivo*. Experiments were performed at embryonic and larval stages, during which sex cannot be reliably determined; therefore, both sexes were included without distinction. Embryos/larvae were obtained from healthy adult breeders and staged by standard criteria (hpf/dpf). The experimental unit was a single embryo/larva (or individual fish for adult endpoints). Sample sizes, numbers of independent biological replicates, and statistical methods are reported for each experiment and figure.

Group allocation was performed by randomization, and image-based quantification was conducted blinded to treatment when feasible using coded files. Pre-defined exclusion criteria included unfertilized embryos, severe baseline developmental delay, technical failure of microinjection (xenografts), or non-specific malformations unrelated to treatment; exclusions and final n values are reported in the figure legends. Animal welfare was monitored daily. Larvae were anesthetized for imaging using tricaine (MS-222; 0.016% w/v, Sigma-Aldrich), and humane endpoints were applied for severe distress or moribund status. Euthanasia, when required, was performed using an approved overdose of tricaine followed by a secondary method consistent with institutional guidelines.

#### 2.4.2 Embryotoxicity Assay

Zebrafish embryos at 6 h post-fertilization (hpf) were exposed to serial dilutions of GACC in 24-well plates (n = 10 embryos/group; 3 biological replicates). Each well contained 1 mL embryo medium. Treatments were maintained for 5 days with daily complete medium renewal: the entire 1 mL was gently aspirated from the edge of the well (avoiding contact with embryos) and replaced with 1 mL of freshly prepared, concentration-matched dosing solution using a P1000 pipette. The vehicle control contained 0.1% (v/v) DMSO; under our experimental conditions, 0.1% DMSO did not produce detectable differences in survival or morphology between 0% and 0.1% DMSO groups under our conditions. Survival, hatching, and gross morphology compared with 0% DMSO controls.

Endpoints were predefined and recorded daily, including (i) survival, (ii) hatching rate, and (iii) morphological abnormalities scored as present/absent for each embryo: developmental delay, body-axis curvature, pericardial edema, yolk-sac edema, craniofacial malformations, tail malformations, and impaired swimming/touch response. Median lethal concentration (LC_50_; based on mortality) and median effective concentration (EC_50_; based on the proportion of embryos displaying any predefined abnormality) were calculated by nonlinear regression using a four-parameter logistic (4PL) model [log(concentration) vs. response, variable slope] in GraphPad Prism v10.0 (GraphPad Software). LC_50_/EC_50_ values are reported with 95% confidence intervals; goodness of fit was evaluated by R^2^ and inspection of residuals.

#### 2.4.3 Hepatotoxicity (Larval Liver Readouts)

For liver safety assessment, *Tg (fabp10a:EGFP-mCherry)* larvae were exposed to GACC from 2 to 5 post-fertilization (dpf). The final DMSO concentration was 0.1% (v/v) in all treatment and vehicle solutions. Each experiment included a vehicle-only (0.1% DMSO) control group; under our conditions, 0.1% DMSO did not measurably alter liver fluorescence compared with 0% DMSO controls.

Larvae were anesthetized with tricaine (0.016% w/v; MS-222, Sigma-Aldrich) and positioned laterally in embryo medium for imaging. Fluorescence images were acquired using an ImageXpress^®^ Micro High-Content Imaging System (Molecular Devices, San Jose, CA, USA). with a 4× objective (single-field liver capture). EGFP and mCherry signals were collected using the instrument’s GFP and Texas Red/mCherry filter sets. Exposure time, gain, and illumination intensity were determined using vehicle controls to avoid signal saturation and were kept constant across all groups within each experiment; images were acquired in the same bit depth and resolution settings throughout.

Liver regions of interest (ROIs) were defined in Fiji/ImageJ (v2.16.0; National Institutes of Health [NIH], Bethesda, MD, USA) by outlining the liver boundary on the merged EGFP/mCherry image (fabp10a-positive area), with background subtraction performed using an adjacent non-fluorescent region of the same larva. Liver area and mean fluorescence intensity (MFI) were then quantified from the ROI, and values were normalized to body length to control for size differences. Hepatotoxicity was defined a priori as a **≥**20% reduction in liver area or MFI vs. vehicle, or the presence of liver atrophy or hemorrhage. Image files were coded prior to analysis, and ROI drawing/quantification was performed in a blinded manner. Per-larva data were averaged within each biological replicate, and group means were compared to vehicle controls.

#### 2.4.4 Xenograft Assay

Hep3B cells were labeled with Vybrant^™^ DiI cell-labeling solution (cat. no. V22885; Thermo Fisher Scientific/Invitrogen, Waltham, MA, USA) according to the manufacturer’s instructions. Cells were counted and resuspended at a defined concentration to deliver a consistent inoculum per embryo, and 200 Dil-labeled cells in 4.6 nL were microinjected into the yolk sac of 2 dpf embryos using a pneumatic microinjector and pulled glass capillaries. Injection volume was calibrated before each session using a micrometer scale, and the target cell number was controlled by the pre-set cell concentration and fixed injection volume. Injection success was verified by immediate visualization of localized DiI fluorescence at the yolk sac, and only embryos with comparable fluorescence signals and no leakage were included for downstream analyses.

At 1 day post-injection (dpi; 3 dpf), embryos with detectable xenograft fluorescence were imaged (baseline), randomly allocated to treatment groups, and exposed for 48 h to GACC (50 µM), sorafenib (10 µM; Bayer, Leverkusen, Germany), or vehicle (0.1% DMSO). Tumor burden was assessed again at 3 dpi (5 dpf). Fluorescence images were acquired on a Leica DMIRB inverted microscope (Leica Microsystems, Wetzlar, Germany) equipped with an Olympus DP73 camera (Olympus Corporation, Tokyo, Japan) using identical objective, illumination, and exposure settings across all groups within each experiment.

For quantification, the region of interest (ROI) was defined manually in ImageJ (v2.16.0; NIH) by outlining the DiI-positive xenograft area based on fluorescence boundaries, and integrated density (integrated fluorescence intensity) was measured after background subtraction using an identical workflow for all embryos. To normalize for baseline variability in injection size, tumor burden was expressed as the change in integrated density from 1 dpi to 3 dpi and/or as 3 dpi signal normalized to the corresponding 1 dpi value for each embryo (as specified in the figure legend). Image quantification was performed in a blinded manner with respect to treatment group. Under these conditions, no significant differences in survival or gross morphology were observed between 0% and 0.1% DMSO controls.

#### 2.4.5 Liver Tumor Histology

Liver tumor histology was performed using the *Tg(fabp10a:tert)* transgenic zebrafish line and the HSPR line (*fabp10a*-driven *HBx*, *src*, *RPIA* on a *tp53* mutant background), as previously described [28,29,31]. Fish were maintained under the husbandry conditions specified in Section 2.4.1.

For *Tg(fabp10a:tert)* fish, larvae were exposed to drug (or vehicle) by immersion from 5 to 15 days post-fertilization (dpf). Larvae were euthanized at 15 dpf, and whole larva were fixed for histological analysis. For HSPR line, adults were treated starting at 4 months of age by oral gavage twice weekly for 4 weeks with drug or vehicle, following our published zebrafish gavage procedure [32]. Fish were euthanized at 5 months of age, and livers were dissected immediately for histological analysis.

All samples were processed in parallel using an identical workflow to minimize technical variation. Whole larvae (15 dpf) or dissected adult livers were fixed in 4% paraformaldehyde (PFA) (manufacturer and catalog number listed in Table S1) at 4°C overnight, dehydrated through graded ethanol, paraffin-embedded, and sectioned at 5 µm. Hematoxylin–eosin (H&E) staining was performed by NHRI Pathology Core Facility using standardized reagents and procedures (reagent details provided in Table S1).

Slides were evaluated by a pathologist blinded to group allocation. Histopathological endpoints included: mitotic figures (cells with clearly condensed chromosomes consistent with mitosis), trinucleated hepatocytes (hepatocytes containing three distinct nuclei within a single cell boundary), karyomegaly (enlarged hepatocyte nuclei relative to surrounding hepatocytes), and architecture distortion (disrupted hepatic cord organization and/or abnormal parenchymal architecture), following previously published criteria [33]. Quantitation was performed using 3–5 non-overlapping high-power fields (HPFs) per liver (n = 10 fish/group), and values were averaged per fish prior to group-level statistical comparisons.

### 2.5 Quantitative PCR (qPCR)

Total RNA was isolated from cultured cells or pooled zebrafish livers using the NucleoSpin^®^ RNA Midi kit (MACHEREY-NAGEL, 740962, Düren, Germany), followed by on-column DNase digestion with the rDNase Set (MACHEREY-NAGEL, 740963). RNA concentration and purity were assessed by spectrophotometry, and the same amount of input RNA (1 µg) was used for all samples.

Complementary DNA (cDNA) was synthesized from 1 µg RNA using the iScript^™^ cDNA Synthesis Kit (Bio-Rad Laboratories, 1708891, Hercules, CA, USA). Reverse transcription was performed in a single batch for all samples within each experiment to minimize technical variation.

Quantitative polymerase chain reaction (qPCR) was performed using Fast SYBR^™^ Green Master Mix (Applied Biosystems, 4385612, Waltham, MA, USA) on an Applied Biosystems^™^ ViiA^™^ 7 Real-Time PCR System). Thermal cycling conditions were: 95°C for 3 mins (initial denaturation), followed by 40 cycles of 95°C for 1 s and 60°C for 20 secs. A melting-curve analysis was performed at the end of each run to confirm single specific amplicons. No-template controls and no-reverse-transcription controls showed no amplification.

Each biological sample was analyzed with three technical replicate wells, and ≥3 independent biological replicates were included per condition, as specified in the figure legends. For statistical analyses, technical replicates were averaged to yield a single value per biological replicate. Relative gene expression was normalized to β-actin and calculated by the 2^−ΔΔCt^ method. Primer sequences are provided in Table 1.

**Table 1:** Primer sequences used for qPCR analysis, silver staining/transfection, and DNA polymerase stop assay.

name	Sequences
*ccne1*-F	^5′^TCCCGACACAGGTTACACAA^3′^
*ccne1*-R	^5′^TTGTCTTTTCCGAGCAGGTT^3′^
*cdk1*-F	^5′^CTCTGGGGACCCCTAACAAT^3′^
*cdk1*-R	^5′^CGGATGTGTCATTGCTTGTC^3′^
*cdk2*-F	^5′^CAGCTCTTCCGGATATTTCG^3′^
*cdk2*-R	^5′^CCGAGATCCTCTTGTTTGGA^3′^
*ccnd1*-F	^5′^TTGCCTCTCATCCCAGAACCT^3′^
*ccnd1*-R	^5′^CCTGACACGATCGCAGACAGT^3′^
*myca*-F	^5′^CACGCTGAAAGGAAGGAACTG^3′^
*myca*-R	^5′^GAGGTGCTCAGATCCTGCAAA^3′^
*mycb*-F	^5′^GGTGTTTCCCTTTCCACTGA^3′^
*mycb*-R	^5′^TTCTCTTTTCCACCGTGACC^3′^
β-*actin*-F	^5′^CTCCATCATGAAGTGCGACGT^3′^
β-*actin*-R	^5′^CAGACGGAGTATTTGCGCTCA^3′^
*KRAS-G4*	^5′^TGAGGGCGGTGTGGGAATAGGGAA^3′^
*cy5-KRAS-G4*	^5′^cy5-TGAGGGCGGTGTGGGAATAGGGAA^3′^
*FAM-KRAS*	^5′^FAM-TAATACGACTCACTATAGCAATTGC^3′^
*KRAS-long*	^5′^TGAATCCTGAGGGCGGTGTGGGAAGAGGGAAGATAGCTGCACGCAATTGC TATAGTGAGTCGTATTA^3′^

### 2.6 G-Quadruplex Binding/Stabilization Assays

#### 2.6.1 Native Polyacrylamide Gel Electrophoresis (PAGE) and Silver Staining

An oligonucleotide encompassing the *KRAS* promoter G-quadruplex (G4) motif (Table 1) was annealed under K^+^-containing conditions and the co-incubated with GACC or pyridostatin (PDS). Complexes were incubated for 1 h at 4°C and immediately mixed with native loading dye. Samples were resolved on 16% native polyacrylamide gels containing 12.5 mM KCl, using 12.5 mM KCl as the running buffer, at 100 V, 4°C, for 150 mins.

After electrophoresis, gels were processed by silver staining (standard protocol). Band development was monitored and stopped once bands were clearly visible to avoid signal saturation. Bands intensities were quantified in ImageJ (v2.16.0; NIH) using identical regions of interest (ROIs) with background subtraction, and only non-saturated bands were used for densitometric comparisons. Quantification was repeated independently to assess technical variability. Controls included DNA-only (no ligand; 0 condition), ligand-only, and non-G4/mutant oligonucleotide where indicated.

#### 2.6.2 DNA Polymerase Stop Assay

DNA polymerase stop assay was performed following the protocol by Wang et al. [34]. A Fluorescein (FAM)-labeled KRAS oligonucleotide (FAM-KRAS) was annealed to the complementary KRAS-long strand at a 1.2:1 molar ratio (120 µL 1 µM FAM-KRAS + 100 µL 1 µM KRAS-long), with KRAS-long as the limiting strand, to drive complete duplex formation. to yield a 1 µM duplex. The annealing mixture contained a maximum of 100 pmol duplex in 220 µL (final duplex concentration ~0.455 µM), and duplex DNA was used based on the concentration defined by the limiting strand. Annealing conditions were 95°C for 5 min followed by slow cooling to room temperature.

For ligand binding, 20 µL of annealed duplex (~0.455 µM) was mixed with 20 µL ligand solution (GACC 25–100 µM or PDS 0.75–3 µM) and incubated for 3 h at room temperature, as described in Wang et al. [34], yielding a final duplex concentration of ~0.227 µM. Primer extension reactions were initiated by adding Taq DNA polymerase (Invitrogen, 11615-010), the supplied buffer, Mg^2+^, and dNTPs, followed by incubation at 72°C for 30 min (final concentration: 0.1 mM dNTPs, 2 mM MgCl_2_, 50 mM KCl, and 1.25 U/μL Taq DNA polymerase). Samples were then mixed with 10 µL formamide-based denaturing loading dye and heat-denatured immediately before loading to prevent strand re-annealing.

Extension products were resolved on 12% denaturing polyacrylamide gels (8 M urea) in 1× Tris-borate-EDTA (TBE) buffer. For a 15 mL gel solution, 1.5 mL 10× TBE, 6 mL 30% acrylamide:bis (29:1), 7.2 g urea (to 8 M), and deionized water to 15 mL were combined. The solution was stirred vigorously to fully dissolve urea, then 15 µL N,N,N^′^,N^′^-tetramethylethylenediamine (TEMED) and 150 µL fresh 10% (w/v) ammonium persulfate (APS) were added, mixed, poured, and allowed to polymerize for ~30 min. Gels were run at 100 V (13–20 mA) for ~3 h at room temperature, until baseline separation of size-differing products was achieved.

Bands were visualized via the FAM signal (Alexa Fluor 488channel), and band intensities were quantified in ImageJ (v2.16.0; NIH) using identical ROIs and background subtraction across samples, with imaging performed under identical acquisition settings and only non-saturated bands used for densitometry. Controls included DNA-only, ligand-only, and, where indicated, non-G4/mutant duplex.

#### 2.6.3 G4 Immunofluorescence and KRAS Transcription Assay

Hep3B cells were transfected with Cy5-labeled KRAS-G4 oligonucleotides (MDBio, Inc., Taipei, Taiwan) using Lipofectamine 3000 (Invitrogen, L3000001) according to the manufacturer’s protocol, and then treated with GACC (200 µM) or pyridostatin (PDS; 10 µM; Selleck Chemicals, S7444) for 24 h. Cells were fixed in 4% paraformaldehyde, permeabilized with 0.2% Triton X-100, and blocked with 5% bovine serum albumin (BSA; Sigma-Aldrich, A9647). To visualize G4 structures, cells were incubated with BG4 single-chain variable fragment (scFv, FLAG-tagged, Absolute Antibody, Ab00174-1.1; Oxford, UK), followed by rabbit anti-DYKDDDDK (FLAG) antibody (Cell Signaling Technology, #2368; Danvers, MA, USA, 1:500) which recognizes the same epitope as the Sigma-Aldrich Anti-FLAG M2 antibody. Fluorescent detection used goat anti-rabbit IgG (H+L), DyLight 488 (GeneTex, GTX213110-04, Hsinchu, Taiwan, 1:500). Nuclei were counterstained with DAPI (Invitrogen, D1306) for 1–5 min at room temperature in the dark.

For immunofluorescence quantification, nuclear regions of interest (ROIs) were defined based on DAPI-positive nuclei in MetaMorph (version 7.10.1.161, Molecular Devices, San Jose, CA, USA; RRID:SCR_002368) using a fixed intensity-threshold segmentation workflow with minimal manual correction to exclude overlapping or partial nuclei; measurements were performed by a blinded assessor. The number of cells (nuclei) analyzed per condition is reported in the corresponding figure legend. Co-localization between Cy5-labeled KRAS-G4 oligonucleotides and BG4 staining was assessed qualitatively by fluorescence microscopy and was not quantified using co-localization metrics (e.g., Pearson’s or Manders’ coefficients). *KRAS* mRNA levels in PLC/PRF/5 cells were measured by qPCR as in Section 2.5.

### 2.7 AI-Phenotypic Response Surface (AI-PRS) Optimization

Drug-response surfaces were modeled using our in-house Artificial Intelligence–Phenotypic Response Surface (AI-PRS) pipeline implemented in Google Colaboratory (Python 3; Google LLC, Mountain View, CA, USA). A 3 × 3 × 3 dose matrix was tested for GACC (9.54, 28.61, 85.85 µM), metformin (0.85, 2.55, 7.65 mM), and regorafenib (0.5, 1.5, 4.5 µM). CCK-8 viability based on 3 independent biological replicates.

**Antimicrobial assay:** Antimicrobial testing was conducted by SGS Taiwan Ltd. (New Taipei City, Taiwan) according to USP–NF <51> Antimicrobial Effectiveness Testing. A 0.1 M GACC stock solution was provided, and SGS prepared a 500-fold dilution for testing (final concentration 200 µM). Test organisms included methicillin-resistant *Staphylococcus aureus (MRSA), Klebsiella pneumoniae, Escherichia coli, Pseudomonas aeruginosa, Candida albicans,* and *Candida auris*. Viable counts were determined at 8, 24, and 48 h by serial dilution in SCDLP followed by plating on TSA, with incubation at 30°C–35°C for 3–5 days. Antimicrobial activity was reported as percent reduction in viable counts relative to the initial inoculum, as defined in the USP–NF <51> test report.

### 2.8 Quantification and Statistical Analysis

Data are presented as mean ± standard error of the mean (SEM). n denotes biological replicates; technical replicates (e.g., wells in qPCR and CCK-8 assays) were averaged to a single value per biological replicate. Image quantification (e.g., liver area, fluorescence integrated density) was performed in ImageJ (v2.16.0, NIH) using identical exposure and analysis settings across groups. Dose–response curves and IC_50_ values were estimated by four-parameter logistic (4PL) regression. Normality was assessed by Shapiro–Wilk test. For two-group comparisons, two-tailed unpaired *t*-tests (or Mann–Whitney for non-normal data) were used, for multiple groups, one-way ANOVA with Tukey’s post hoc test was applied. A *p* < 0.05 was considered statistically significant. Statistical analyses were conducted in GraphPad Prism (version 10; GraphPad Software). Exact n values, statistical tests, and *p*/q values are reported in the figure legends.

## 3 Results

### 3.1 Structural Characterization of GACC

**Figure 1:**
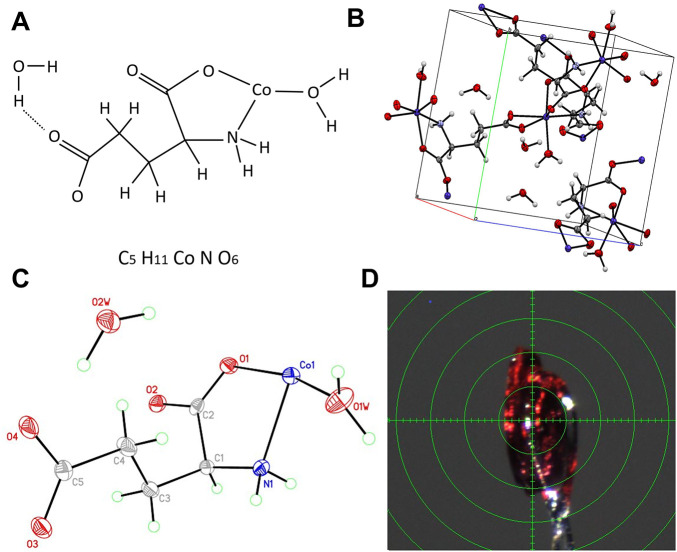
Structural characterization of glutamic acid chelated cobalt (GACC). (**A**) Chemical structure of GACC, drawn using Microsoft PowerPoint (Microsoft Corporation, Redmond, WA, USA). (**B**) Single-crystal X-ray diffraction–derived molecular packing and coordination geometry of GACC, visualized using the Mercury program (version 3.8; Cambridge Crystallographic Data Centre, Cambridge, UK). (**C**) ORTEP diagram of GACC showing thermal ellipsoids at the 50% probability level, generated using the XP program (Bruker AXS, Bruker Corporation, Billerica, MA, USA). (**D**) Crystallographic structure confirming the orthorhombic crystal system. Structural analysis through X-ray diffraction (XRD) and ORTEP representation confirms the stable coordination of cobalt(II) with glutamic acid, forming a robust chelated complex essential for its biological activity.

Glutamic acid–chelated cobalt (GACC) is a cobalt(II) coordination complex with the empirical formula C_5_H_11_CoNO_6_ and a molecular weight of 240.08 g/mol. Single-crystal X-ray diffraction (SC-XRD) showed that GACC crystallizes in an orthorhombic system and adopts a stable planar coordination geometry. ORTEP and packing diagrams confirmed chelation of cobalt by the glutamate carboxylate and amino groups (Fig. 1A–D; Supplementary Tables S2–S6). The defined bond lengths and angles are consistent with a stable coordination environment, supporting the suitability of GACC for downstream biological testing.

### 3.2 GACC Shows Moderate Differential Sensitivity between Hepatoma and Non-Transformed Hepatocytes

**Figure 2:**
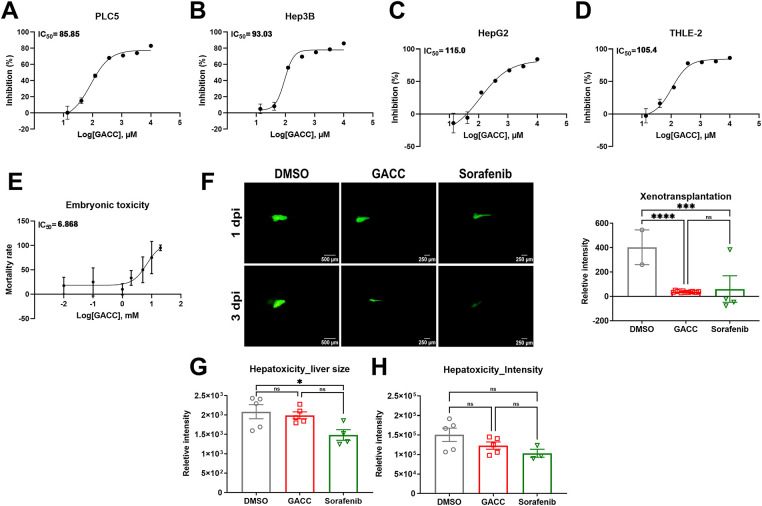
GACC shows modest differential sensitivity *in vitro* and reduces tumor burden in zebrafish with low overt hepatotoxicity readouts. (**A**–**D**) CCK-8 viability assays of PLC/PRF/5 (**A**); Hep3B (**B**); HepG2 (**C**) and THLE-2 (**D**) cells treated with GACC at the indicated concentration for 72 h. Viability is normalized to vehicle control (0.1% DMSO, set to 100%). Data are shown as mean ± SEM from n ≥ 3 independent biological replicates, each measured in triplicate technical wells. IC_50_ values were estimated by 4-parameter logistic regression. (**E**) Zebrafish embryotoxicity dose–response following immersion exposure to GACC beginning at 6 hpf with daily medium renewal for 5 days. Survival/morphology endpoints were used to estimate IC_50_. Data represent mean ± SEM from 3 biological replicates with 10 embryos per concentration per replicate (total embryos indicated in the panel). Vehicle control contained 0.1% DMSO. (**F**) Zebrafish xenograft assay. DiI-labeled Hep3B cells were microinjected into the yolk sac of 2 dpf embryos, followed by immersion treatment starting at 1 dpi with vehicle (0.1% DMSO), GACC (50 µM), or sorafenib (10 µM) for 48 h. Representative fluorescence images (left) and quantification of tumor burden (right) expressed as integrated fluorescence density normalized to vehicle. Data are mean ± SEM; n = 10 embryos per group pooled from 3 independent experiments. Scale bar: top-left and bottom-left 500 µm, all others 250 µm as indicated. (**G**,**H**) Larval hepatotoxicity readouts using Tg(*fabp10a*:EGFP-mCherry) larvae exposed to compounds by immersion from 2 to 5 dpf. (**G**) Quantification of liver area and (**H**) mean fluorescence intensity (MFI) (normalized to body length as described in Methods). Data are mean ± SEM; n = 10 larvae per group from >4 independent experiments. Statistical tests: one-way ANOVA with Tukey’s post hoc test. *p < 0.05, ***p < 0.001, ****p < 0.0001, ns, not significant.

We evaluated the antiproliferative activity of GACC in hepatoma cell lines (PLC/PRF/5, Hep3B, and HepG2) and in the immortalized non-transformed hepatocyte line THLE-2. GACC reduced viability in a dose-dependent manner across all HCC cell lines. The IC_50_ in THLE-2 cells was 105.4 µM, compared with 85.85 µM in PLC/PRF/5 cells and 93.03 µM in Hep3B cells, and was comparable to HepG2 (115.0 µM) (Fig. 2A–D). Thus, GACC exhibited only modest differential sensitivity in this *in vitro* setting (≤1.5-fold across cell lines), indicating limited single-agent selectivity at the cellular level and motivating combination-based approaches.

In an external screening performed by the Development Center for Biotechnology (DCB, Taipei, Taiwan), GACC also inhibited viability across additional cancer cell lines, including U87 glioblastoma (IC_50_ 42 µM), H226 lung cancer (44.5 µM), K562 leukemia (54.5 µM), HeLa cervical cancer (73.7 µM), FaDu hypopharyngeal carcinoma (74.6 µM), and A375 malignant melanoma (102 µM) (Supplementary Table S7).

### 3.3 In Vivo Zebrafish Assessment: Embryotoxicity, Hepatotoxicity Readouts, and Antitumor Activity

To evaluate *in vivo* tolerability, zebrafish embryos were exposed to GACC by immersion over a concentration range for 5 days. The embryotoxicity IC_50_ was 6.868 mM, substantially higher than the therapeutic concentrations used for anticancer testing in cell culture (Fig. 2E), suggesting a preliminary *in vivo* safety margin in this model.

We next tested antitumor activity using a zebrafish xenograft model. Hep3B cells were injected into 2 dpf embryos, which were then treated by immersion with GACC (50 µM) or sorafenib (10 µM). At 48 h post-treatment, GACC reduced xenograft fluorescence by 90% relative to vehicle, whereas sorafenib reduced fluorescence by 85% reduction under matched conditions (Fig. 2F). Both treatments significantly decreased tumor burden compared with vehicle controls.

To assess liver safety, we used *Tg(fabp10a:EGFP-mCherry)* larvae, and quantified liver fluorescence and size under identical imaging settings. GACC did not produce a detectable reduction in liver size or fluorescence intensity compared with vehicle, whereas sorafenib reduced liver size and fluorescence intensity, consistent with hepatotoxic effects in this assay (Fig. 2G,H). We note that these endpoints are morphological/fluorescence readouts and do not substitute for serum biochemistry (e.g., ALT/AST), which is technically challenging in larval zebrafish.

### 3.4 GACC Downregulated Proliferation and **β**-Catenin-Associated Transcripts in Tert Transgenic Zebrafish

**Figure 3:**
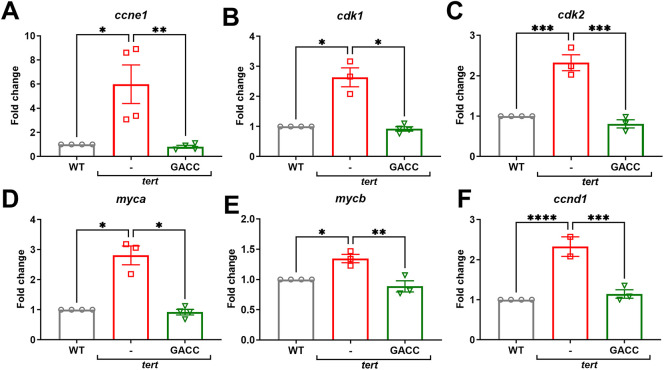

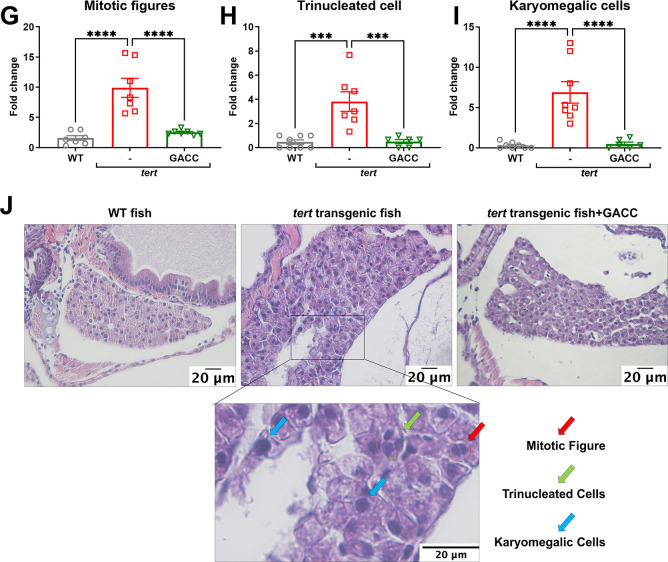
GACC suppresses proliferation-associated and β-catenin-associated transcripts and reduces dysplastic features in *tert* transgenic zebrafish. (**A**–**F**) Quantitative polymerase chain reaction (qPCR) analysis of liver tissue from Tg(*fabp10a*:tert) zebrafish treated by immersion with vehicle (0.1% DMSO) or GACC (50 µM) for 15 days. Relative mRNA expression of cell proliferation markers ((**A**) *ccne1*, (**B**) *cdk1*, (**C**) *cdk2*) and β-catenin-associated target genes ((**D**) *myca*, (**E**) *mycb*, (**F**) *ccnd1*) is shown, normalized to β-actin and expressed relative to vehicle. Data are mean ± SEM; n > 3 biological replicates (each replicate = pooled livers from 10 fish). (**G**–**J**) Liver histopathological in the same treatment groups. (**G**,**H**) Quantification of histologic endpoints including (**G**) mitotic figures, (**H**) trinucleated cells, and (**I**) karyomegalic hepatocytes (scoring method and fields per fish as described in Methods). Data are mean ± SEM; n = 10 fish per group; analysis performed by a blinded assessor. (**J**) Representative H&E-stained sections showing hepatic architecture and cellular atypia. Scale bar: 20 µm. Statistical tests: one-way ANOVA with Tukey’s post hoc test. *p < 0.05, **p < 0.01, ***p < 0.001, ****p < 0.0001.

To examine transcriptional effects *in vivo*, we used liver specific *telomerase reverse transcriptase* (*tert)* overexpressing transgenic zebrafish, which captures early events associated with hepatocarcinogenesis. After 15 days of immersion treatment with GACC (50 µM), qPCR analysis showed significant downregulation of proliferation-related transcripts (*ccne1*, *cdk1*, *cdk2*) and β-catenin-associated target genes (*ccnd1*, *myca*, *mycb*) compared with vehicle controls (Fig. 3A–F).

Histopathological examination of liver sections showed lower rates of mitotic figures (from 9.9% to 2.52%), trinucleated hepatocytes (from 3.81% to 0.5%), and karyomegalic hepatocytes (from 6.88% to 0.5%) after GACC treatment compared with vehicle (Fig. 3G–J), consistent with attenuation of proliferative and dysplastic features at the tissue level in this model.

### 3.5 GACC Is Asscociated with G4 Stabilization and Reduced KRAS Transcription

We next asked whether GACC acts as a G-quadruplex (G4)-interacting compound. Molecular docking predicted favorable binding to multiple G4 targets, including sequences derived from the *KRAS* and *MYC* promoter regions (Table 2).

**Table 2:** Predicting the potential target of GACC by AutoDock.

G4 Target	G4 Type	Binding Energy (kcal/mol)	Inhibition Constant
*TERRA* (PDB ID: 3IBK)	RNA G4	−7.93	1.53 µM
*KRAS* (PDB ID: 6N65)	DNA G4	−7.33	4.22 µM
*MYC* (PDB ID: 6JJ0)	DNA G4	−6.88	9.08 µM
*Telomere* (PDB ID: 6CCW)	DNA G4	−6.65	13.26 µM
*RG-1* (https://reurl.cc/deylK6)	DNA G4	−5.89	48.46 µM
*PDGFR-β* promoter (PDB ID: 6V0L)	DNA G4	−5.47	97.32 µM
*hTERT* promoter (PDB ID: 2KZE)	DNA G4	−5.34	121.06 µM
*hBcl-2* promoter (PDB ID: 2F8U)	DNA G4	−5.25	142.29 µM
*hCKIT-2* promoter (PDB ID: 2KYP)	DNA G4	−5.17	162.44 µM

Although native PAGE with silver staining did not clearly resolve a discrete GACC–G4 complexes under our conditions—despite buffer optimization (12.5 mM KCl in the gel/running buffer; 37.5 mM KCl and 12.5 mM phosphate in the binding mix) and increased KRAS-G4 DNA input to reduce nonspecific bands (Fig. S1A,B)—a DNA polymerase stop assay provided functional evidence of G4-stabilization.

**Figure 4:**
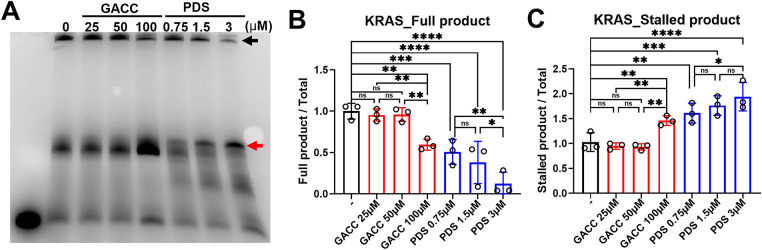

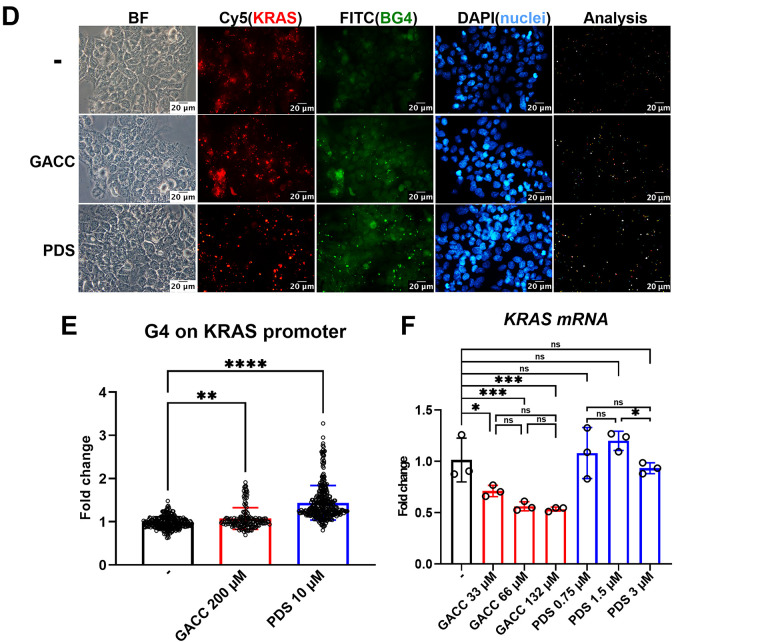
Functional and cellular assays support GACC-associated stabilization of the *KRAS* promoter G-quadruplex and reduced *KRAS* transcription. (**A**–**C**) DNA polymerase stop assay using a KRAS promoter G4-containing template incubated with GACC (25–100 µM) prior to primer extension. (**A**) Representative denaturing PAGE showing full-length and stalled extension. (**B**,**C**) Quantitation of band intensities expressed as % of vehicle control (full-length products and stalled products as indicated), with PDS as a positive control and mutant/non-G4 templates and ligand-only controls included as specificity controls (lanes labeled in the gel). Data are mean ± SEM from n = 3 independent experiments. (**D**,**E**) BG4 immunofluorescence analysis in Hep3B cells transfected with Cy5-labeled *KRAS*-G4 oligonucleotide and treated with vehicle, GACC (200 µM) or PDS (10 µM) for 24 h. (**D**) Representative images showing nuclear BG4 signal (and Cy5/DAPI channels as shown). The Analysis panels are MetaMorph-generated spot/colocalization maps derived from the corresponding images and share the same spatial calibration. Scale bar: 20 µm (applies to all panels in (**D**), including Analysis). (**E**) Quantification of nuclear BG4 intensity per cell (analysis performed using identical thresholds and ROIs across conditions). Data are mean ± SEM; hundreds of cells pooled from 3 independent experiments. (**F**) *KRAS* mRNA levels in PLC/PRF/5 cells treated with GACC and PDS at the indicated concentration for 24 h, measured by qPCR and normalized to β-actin, expressed relative to vehicle. Data are mean ± SEM; n ≥ 3 biological replicates. Statistical tests: one-way ANOVA with Tukey’s post hoc test. *p < 0.05, **p < 0.01, ***p < 0.001, ****p < 0.0001; ns, not significant.

Pre-incubation of annealed *KRAS* G4 templates with GACC (25–100 µM) produced dose-dependent stalling at predicted G4 positions within the *KRAS* promoter, reflected by increased truncated extension products (Fig. 4A–C). Pyridostatin (PDS) served as a positive control and generated the expected strong stop signals, whereas non-G4/mutant templates and ligand-only controls did not yield specific stall bands. Together, these findings indicate that primer-extension assays are more sensitive than native gels for detecting GACC-associated stabilization of KRAS G4 motifs in this system.

Consistent with these results, immunofluorescence in Hep3B cells transfected with Cy5-labeled *KRAS*-G4 oligomers showed increased nuclear G4 signal after GACC treatment, comparable to PDS (Fig. 4D,E). GACC also increased G4 signal in the RG1 region (Fig. S2). In PLC/PRF/5 cells, GACC significantly reduced KRAS mRNA abundance by qPCR (Fig. 4F). These results support the hypothesis that GACC stabilizes G4 structure, thereby suppressing oncogenic transcription. Collectively, polymerase-stop stalling at the *KRAS* promoter G4 motif, increased nuclear BG4 signal, and reduced *KRAS* transcript levels support a G4-associated mechanism, although we cannot exclude contributions from non-G4 pathways or from RNA G4 structures under our experimental conditions.

### 3.6 Exploratory Antiviral and Antimicrobial Testing (Supplementary)

Because GACC was originally developed outside oncology, we performed limited exploratory assays against selected viral and microbial targets. *In vitro*, GACC reduced influenza H1N1 and H3N2 plaque formation with apparent IC_50_ values of 5.241 and 3.447 mM, respectively (Fig. S3).

In standardized broth-microdilution tests conducted by SGS Taiwan, GACC inhibited growth of MRSA, *Pseudomonas aeruginosa*, *Klebsiella pneumoniae*, and *Escherichia coli* by ≥97% within 48 h and showed antifungal activity against *Candida albicans* (92.7%) and *Candida auris* (88.2%) (Supplementary Table S8). These findings are preliminary and are provided in the Supplementary Information and concentration ranges; they are not central to the HCC-focused conclusions of this study.

### 3.7 Synergistic Inhibition of HCC by GACC, Metformin, and Regorafenib

**Figure 5:**
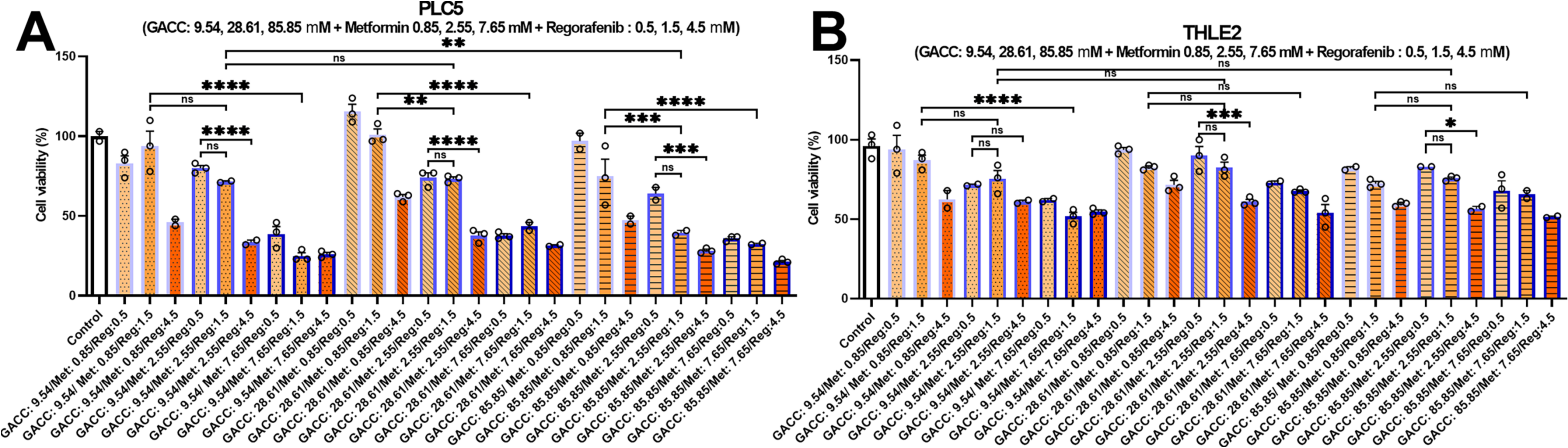

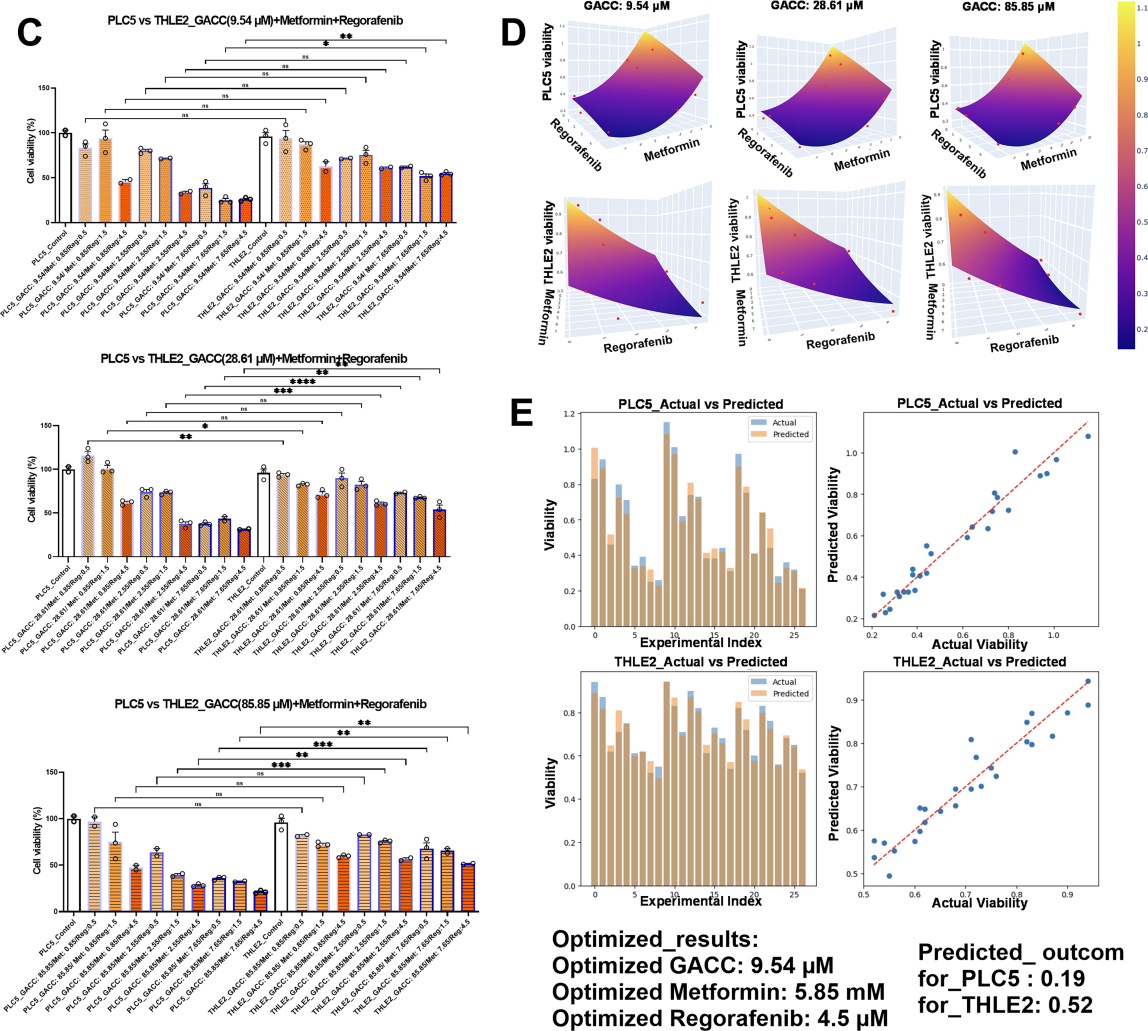
(AI-PRS optimization of combination therapies: GACC, metformin, and regorafenib. (**A–C**) Viability responses for the tested 3 × 3 × 3 dose grid of GACC (9.54, 28.61, 85.85 µM), metformin (0.85, 2.55, and 7.65 mM), and regorafenib (0.5, 1.5, 4.5 µM) in hepatoma cells (PLC5) and non-transformed hepatocytes (THLE-2), as indicated. (**D**) AI-phenotypic response surface (AI-PRS) fit across the dose grid used to identify dose regions with improved efficacy–toxicity balance. (**E**) Observed vs. AI-PRS–predicted viability for the off-grid optimal dose combination in PLC/PRF/5 and THLE-2. Data are presented as mean ± SEM; n values and statistical methods are provided in Section 2.8. Statistical annotations: ns, not significant; *p < 0.05; **p < 0.01; ***p < 0.001; ****p < 0.0001.)

We next tested whether GACC could enhance the activity of established HCC therapeutics. Using our AI-guided phenotypic response surface (AI-PRS) workflow, we modeled 27 (3^3^) GACC–metformin–regorafenib combinations across a dose grid (Fig. 5A–C), and fit a response surface (Fig. 5D). AI-PRS identified an off-grid predicted optimum—GACC 9.54 µM, metformin 5.85 mM, and regorafenib 4.5 µM—which was then confirmed experimentally (Fig. 5E). Under this regimen, PLC/PRF/5 viability decreased to 19%, whereas THLE-2 viability remained 52% (Fig. 5E), indicating improved differential activity compared with single-agent or doublet conditions tested at matched dose levels (Fig. 5A–E). Results were reproducible across independent experiments (n values reported in legends), and statistical testing was performed as described in Section 2.8.

**Figure 6:**
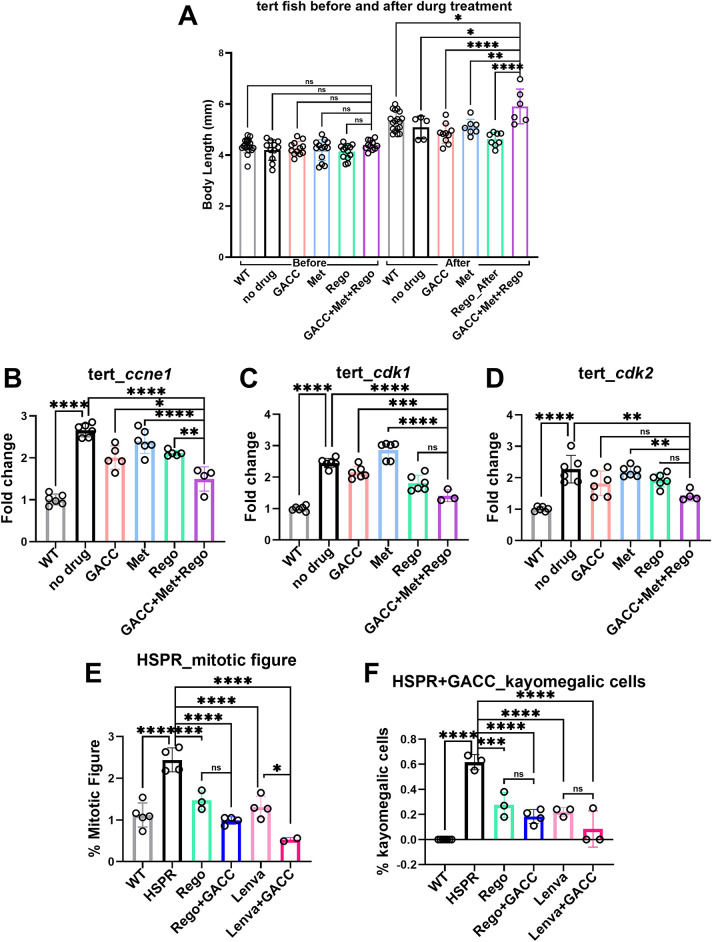
Combination therapies of GACC, metformin, and regorafenib for Hepatocellular Carcinoma (HCC) treatment in *tert* transgenic zebrafish larvae and HBx, src, p53-, RPIA (HSPR) adult zebrafish model. (**A**) Evaluation of drug-induced side effects by measuring body length in tert transgenic zebrafish larvae before and after treatment, assessing potential toxicity and systemic impact. (**B**–**D**) qPCR analysis of cell proliferation markers (*ccne1*, *cdk1*, *cdk2*) in tert transgenic zebrafish larvae, demonstrating significant downregulation following combination therapy. (**E**,**F**) Histological examination of liver cancer hallmarks in HSPR transgenic zebrafish using hematoxylin and eosin (H&E) staining, revealing a significant reduction in mitotic figures, karyomegalic cells, and hepatocellular dysplasia following combination therapy. n = 10 embryos/group; one-way ANOVA with Dunnett’s post-hoc vs. vehicle; *p* thresholds as shown. *p < 0.05, **p < 0.01, ***p < 0.005, ****p < 0.001, ns, not significant.

In zebrafish this combination preserved developmental endpoints, including body length, while reducing expression of proliferation/cell-cycle–associated transcripts (*ccne1*, *cdk1*, and *cdk2*) (Fig. 6A–D), supporting *in vivo* activity with low overt toxicity in this model. In the HSPR transgenic zebrafish model (*fabp10a*-driven HBx, src, and RPIA on a tp53 mutant background), GACC combined with either regorafenib or lenvatinib reduced histological features associated with malignancy, including the proportions of mitotic figures and karyomegalic cells (Fig. 6E,F).

## 4 Discussion

This study characterizes glutamic acid–chelated cobalt (GACC) as a structurally distinct cobalt–glutamate coordination complex with G4-associated activity antitumor effects in HCC models. By combining cell-based viability assays, zebrafish embryo and liver readouts (immersion exposure), xenograft efficacy testing, and mechanistic assays (polymerase-stop stalling, BG4 immunofluorescence, and KRAS transcript measurements), we provide convergent evidence consistent with a mode of action involving stabilization of G-quadruplex (G4) structures and repression of oncogenic transcription. In parallel, an AI-guided phenotypic response surface (AI-PRS) workflow identified a combination regimen with improved differential activity relative to single agents. Together, these data position GACC as a candidate scaffold for further optimization and mechanistic validation in the context of HCC.

### 4.1 GACC as a Structurally Distinct, G4-Associated Compound

Multiple assays in this study support a G4-associated mechanism for GACC. GACC produced dose-dependent polymerase stalling at a *KRAS* promoter G4 motif, increased nuclear BG4 signal following GACC treatment, and reduced *KRAS* mRNA levels. Taken together, these results are consistent with G4 stabilization contributing to transcriptional repression.

GACC differs structurally from classical aromatic G4 ligands, such as TMPyP4, BRACO-19, and CX-5461, While these agents established proof-of-concept for targeting G4 biology, their translational progress has been limited by issues including off-target binding (e.g., duplex DNA intercalation), systemic toxicity, and dose-limiting adverse effects [20,21,35]. In contrast, GACC is a small coordination complex built on an amino-acid backbone, which may confer different physicochemical and cellular handling properties. However, our data do not yet establish that GACC is a selective G4 ligand in the strict sense.

Importantly, we did not perform key experiments required to demonstrate causal specificity for G4 stabilization, including: (i) non-G4 or G4-disrupting sequence controls in all assays, (ii) BG4 competitionexperiments, (iii) RNase treatment to evaluate potential contributions from RNA G4 structures, and (iv) orthogonal biophysical binding measurements (e.g., CD melting, fluorescence titration, ITC, SPR). These assays will be prioritized in future work to strengthen mechanistic attribution and define binding preference and affinity.

### 4.2 A Transporter-Mediated Uptake/Retention Model Remains Hypothetical

We previously referred to a “Nitrogen Trap” concept to explain potential tumor-biased retention of GACC. Given Reviewer concerns, we clarify that this model is hypothesis-generating rather than evidence-based in the current study. Many tumors, including HCC, upregulate amino-acid and anion transporters (e.g., SLC1A5/ASCT2, SLC7A11/xCT, and SLC38A5 [36–38]), raising the possibility that a glutamate-containing chelate could exhibit transporter-dependent uptake or retention in malignant cells. However, we did not measure transporter expression, uptake kinetics, intracellular cobalt speciation, or transporter dependence.

Future studies will directly test this hypothesis by quantifying cellular uptake (e.g., ICP-MS for cobalt), evaluating transporter inhibition/knockdown, assessing pH dependence of intracellular accumulation, and mapping subcellular localization. These experiments will also help determine whether putative transporter-mediated entry is required for downstream G4-associated effects on transcription.

### 4.3 Zebrafish In Vivo Data: Efficacy with Defined Limitations

Zebrafish enabled rapid *in vivo* assessment of efficacy and tolerability using standardized immersion exposure. At concentrations effective in xenograft assays (e.g., 50 µM), GACC reduced tumor-associated fluorescence in Hep3B xenografts and modulated proliferation-related transcripts in a tert-driven liver model. Histopathology further showed reduced rates of mitotic figures, trinucleated hepatocytes, and karyomegalic hepatocytes in the GACC-treated group, consistent with attenuation of proliferative and dysplastic features at the tissue level (Fig. 3G–J).

At the same time, zebrafish readouts have important limitations. First, we did not measure internal exposure (e.g., blood concentration, tissue levels) or absorption kinetics following immersion, which constrains interpretation of dose–exposure relationships. Second, our “hepatotoxicity” assessment relied on liver fluorescence/size and morphology in a transgenic line. These endpoints are useful screening tools but do not substitute for biochemical liver injury markers (e.g., ALT/AST) or histologic necroinflammation scoring. Third, cross-species differences in metabolism and transport mean that mammalian validation will be essential.

Accordingly, we frame the zebrafish data as supportive *in vivo* evidence that motivates follow-up pharmacokinetic, biodistribution, and toxicology studies in mammalian models.

### 4.4 **β**-Catenin–Associated Transcriptional Changes: Association Rather than a Direct G4 Link

In the *tert*-driven zebrafish model, GACC downregulated β-catenin-associated target genes (*ccnd1, myca, mycb*) alongside proliferation markers. These data suggest that GACC impacts transcriptional programs relevant to liver tumor initiation/progression. However, β-catenin pathway modulation can arise from multiple upstream inputs unrelated to G4 stabilization. Our study does not establish a direct mechanistic link between G4 stabilization and β-catenin suppression. Future work will determine whether β-catenin changes occur downstream of KRAS repression, reflect broader stress responses, or involve additional targets.

### 4.5 Combination Optimization and Comparative Context

Single-agent GACC showed only modest differential sensitivity between HCC cells and THLE-2 hepatocytes *in vitro* (≤1.5-fold across tested lines), which does not meet conventional pharmacological standards for selectivity. We therefore emphasize combination optimization as a practical path to improve differential efficacy. Using AI-PRS, we identified a GACC-metformin-regorafenib regimen that substantially reduced PLC/PRF/5 viability while partially preserving THLE-2 viability, and the regimen was tolerated in zebrafish developmental endpoints under the tested conditions. This illustrates how phenotypic modeling can support rational polypharmacy when a single agent has limited selectivity.

Placing these findings into the current HCC therapeutic landscape is also important. Approved systemic therapies such as sorafenib and lenvatinib provide clinical benefit but are constrained by toxicity and acquired resistance. G4 ligands such as PDS (tool compound) and CX-5461 (clinically advanced) demonstrate the potential of G4 biology but also highlight challenges in selectivity and safety. In this context, GACC may represent an alternative scaffold class; however, meaningful benchmarking will require head-to-head comparisons against established G4 ligands (e.g., PDS, CX-5461) and clinically relevant HCC agents using standardized assays and exposure controls.

### 4.6 Exploratory Antiviral and Antimicrobial Findings Should Be Interpreted Cautiously

We observed antiviral and antimicrobial activity of GACC in exploratory assays at high micromolar–millimolar concentrations (Figs. S3 and Table S8). These experiments were not designed to establish mechanism, selectivity indices, or therapeutic relevance, and they are not central to the HCC-focused conclusions of this work. While G4 motifs exist in some microbial and viral genomes [36–38], we did not test whether the observed effects are G4-dependent. We therefore present these data as preliminary observations that may motivate separate future studies, rather than as substantiated therapeutic claims.

### 4.7 Translational Considerations and Key Limitations

Several issues must be addressed before translational development can be considered. First, pharmacokinetic and biodistribution studies are needed to define systemic exposure, liver delivery, clearance, and tissue retention after relevant dosing routes. Second, as a cobalt-containing compound, GACC raises legitimate concerns about long-term metal accumulation and chronic toxicity; these risks should be assessed through tissue cobalt measurements and repeat-dose toxicology in mammals. Third, stronger mechanistic validation is required to establish G4 specificity and causal linkage to KRAS repression. Finally, additional models with higher clinical relevance—such as mammalian HCC models, patient-derived xenografts/organoids, and transcriptomic profiling—will be necessary to define therapeutic scope and identify on-target and off-target pathways.

## 5 Conclusions

GACC is a structurally distinct cobalt–glutamate coordination complex that exhibits antitumor activity in HCC cell lines and zebrafish models and produces molecular changes consistent with G4-associated stabilization at a *KRAS* promoter motif, increased cellular G4 signal, and reduced *KRAS* transcription. While single-agent selectivity *in vitro* is modest, AI-PRS-guided combination optimization identified a regimen with improved differential activity and tolerability in zebrafish screening endpoints. The antimicrobial and antiviral observations are preliminary and are reported as exploratory findings.

Overall, these results support further development of GACC as a candidate scaffold for G4-targeted or G4-associated strategies in HCC, with priority for (i) rigorous G4 specificity testing, (ii) transporter/uptake studies, and (iii) pharmacokinetic and cobalt safety evaluation in mammalian systems.

## Supplementary Materials









## Data Availability

All data supporting the findings of this study are presented within the article and its supplementary materials. Further information and materials are available from the corresponding author upon reasonable request.

## Supplementary Materials

The supplementary material is available online at https://www.techscience.com/doi/10.32604/or.2026.074144/s1.
