# Supplementary figures and images for: Glutamic Acid–Chelated Cobalt Stabilizes G-Quadruplexes and Selectively Suppresses Hepatocellular Carcinoma Growth
Source: Oncol Res. 2026 Mar 23;34(4):21. doi: 10.32604/or.2026.074144 (PMC13040310; doi:10.32604/or.2026.074144)

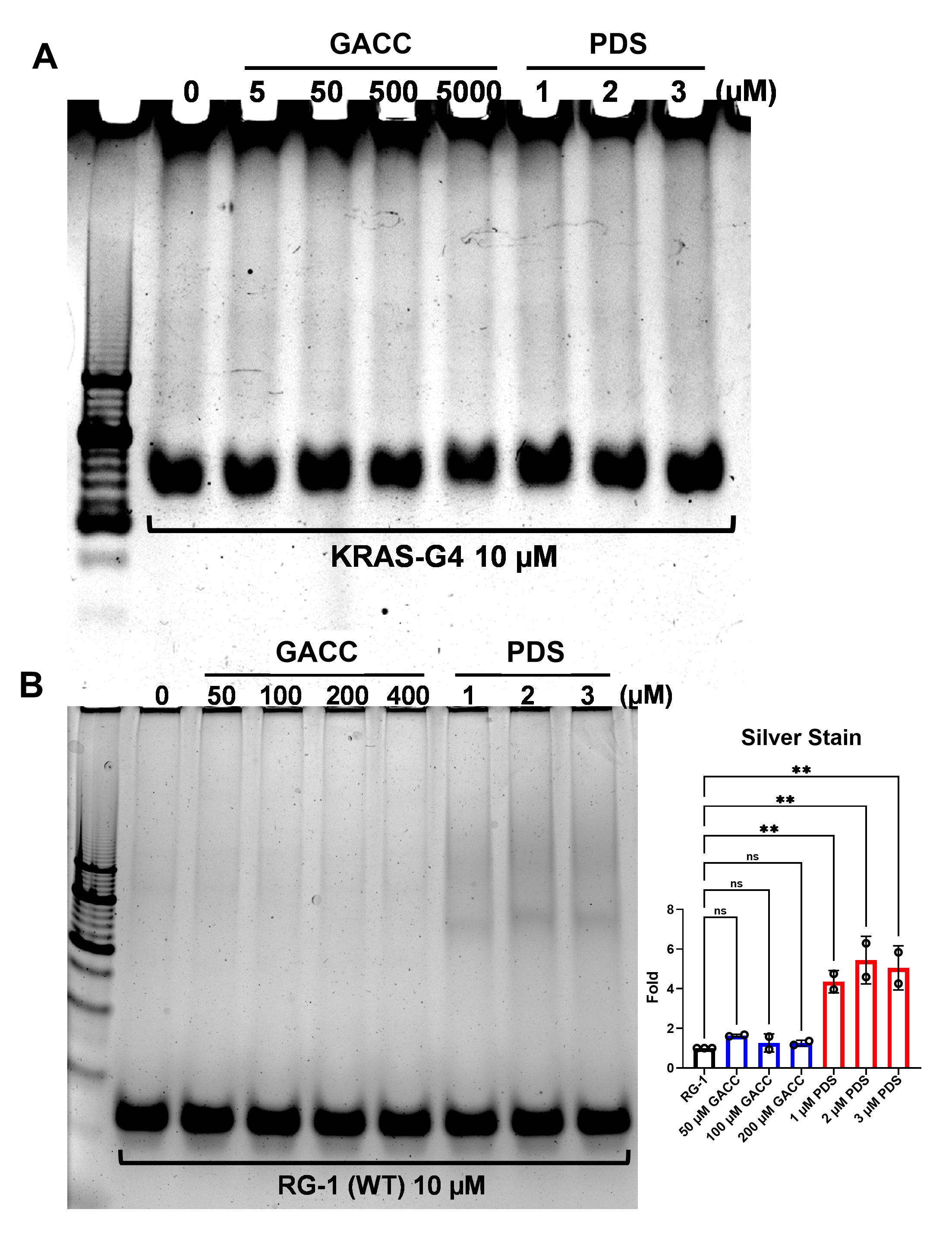

Supplement: Supplementary file 1 [file OncolRes-34-74144-s001.tif]

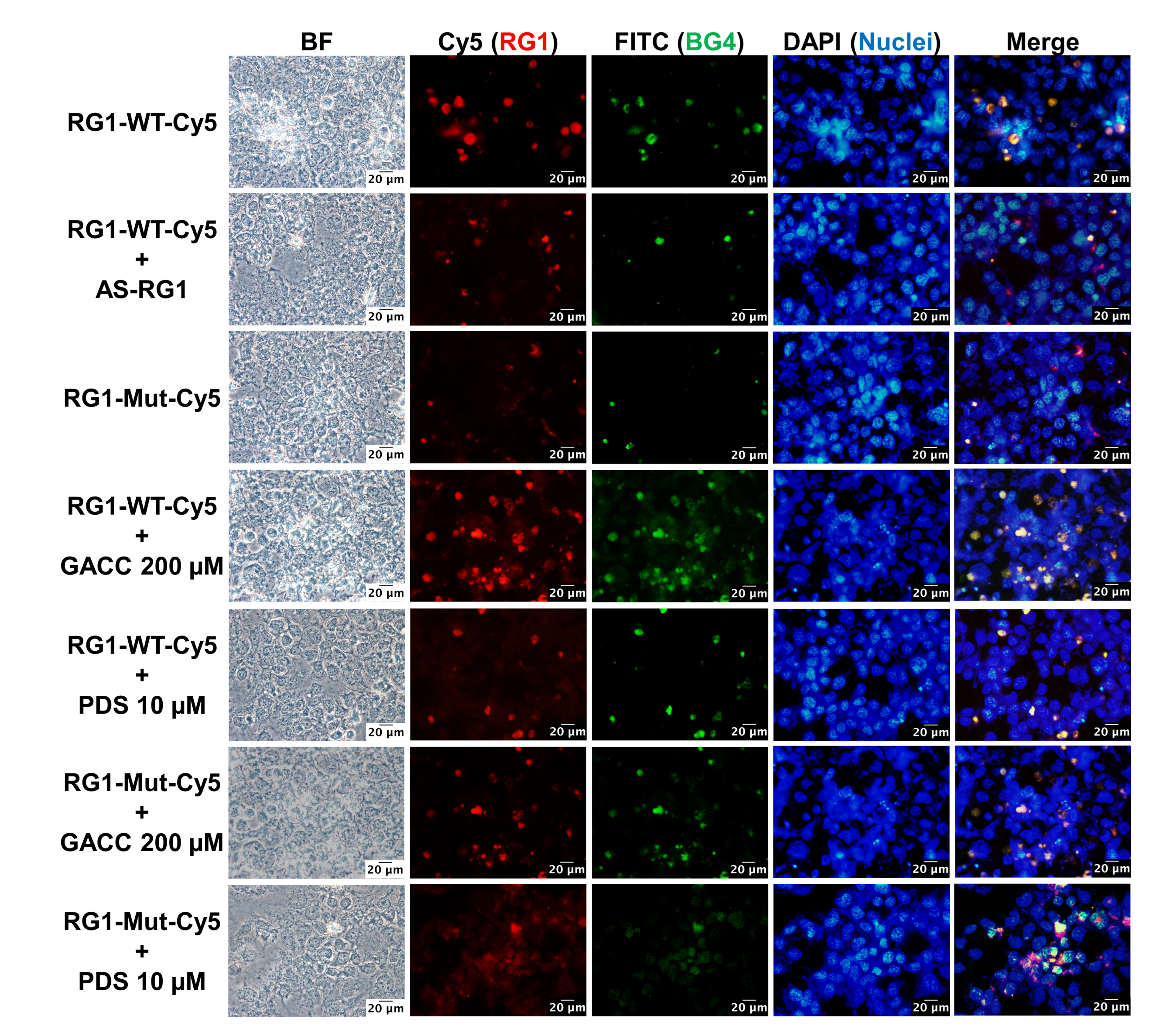

Supplement: Supplementary file 2 [file OncolRes-34-74144-s002.tif]

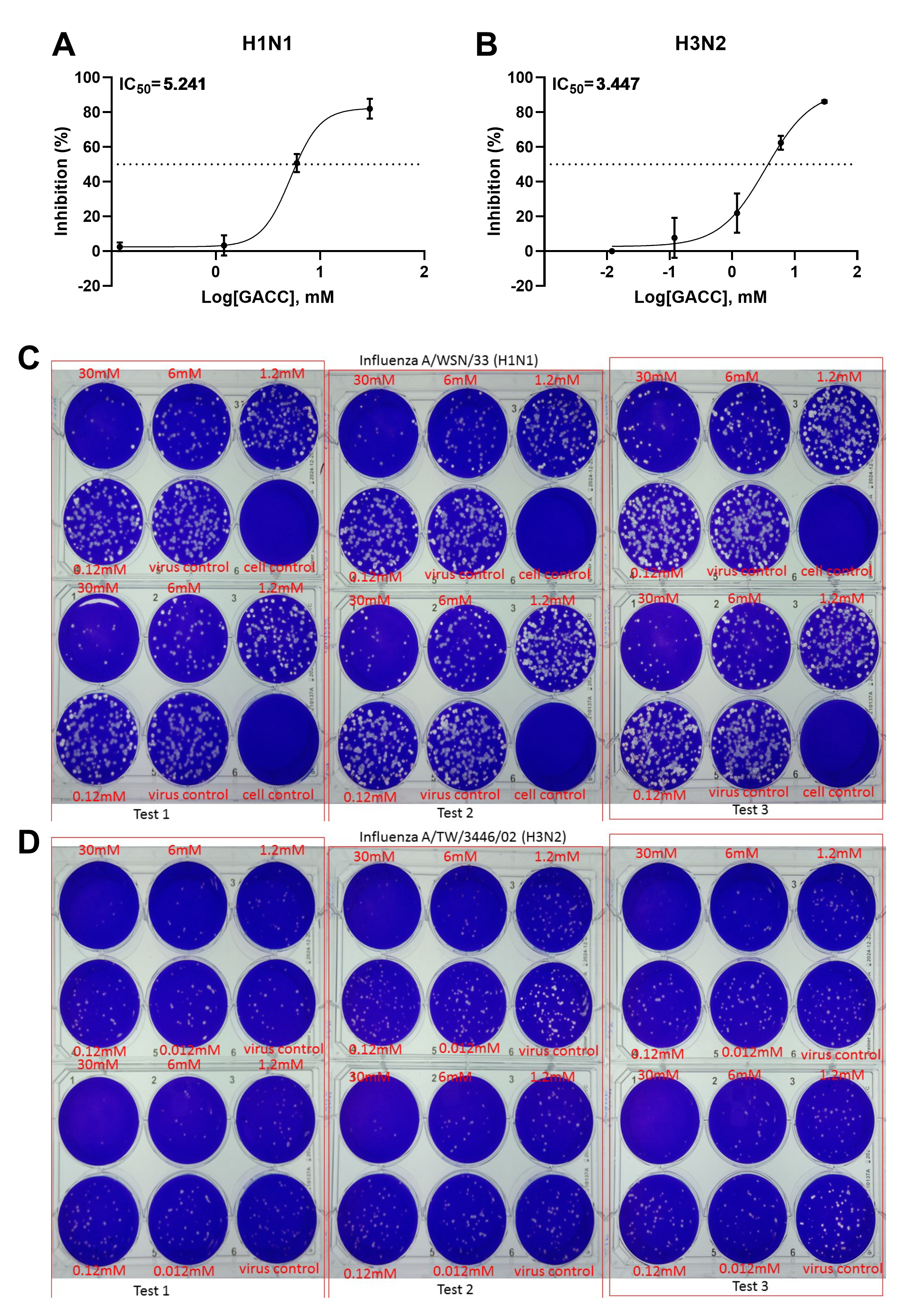

Supplement: Supplementary file 3 [file OncolRes-34-74144-s003.tif]
