# Supplementary material for: Glutamic Acid–Chelated Cobalt Stabilizes G-Quadruplexes and Selectively Suppresses Hepatocellular Carcinoma Growth
Source: Oncol Res. 2026 Mar 23;34(4):21. doi: 10.32604/or.2026.074144 (PMC13040310; doi:10.32604/or.2026.074144)
Supplement: Supplementary file 4 [file OncolRes-34-74144-s004.docx]

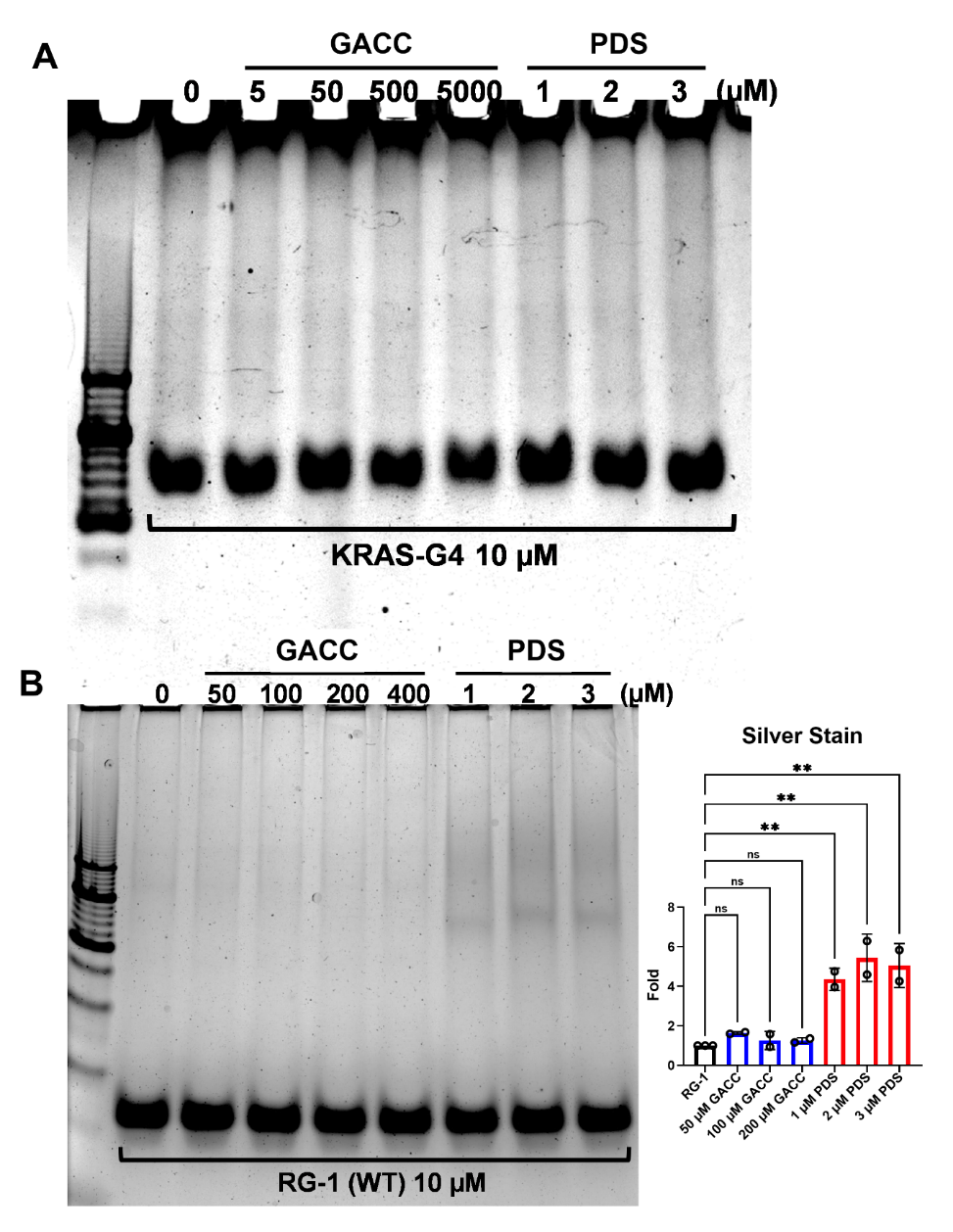
**Figure S1. Native PAGE and silver staining of KRAS-G4 and RG-1 G4 oligonucleotides incubated with GACC or PDS.** (**A**) **KRAS-G4** oligonucleotide (10 µM) incubated with GACC (0, 5, 50, 500, 5000 µM) or pyridostatin (**PDS**, 1–3 µM), resolved by native PAGE and visualized by silver staining. (**B**) **RG-1 (WT)** oligonucleotide (10 µM) incubated with GACC (0, 50, 100, 200, 400 µM) or PDS (1–3 µM), analyzed as in (A). **Right:** densitometric quantification of the RG-1 band intensity from the silver-stained gel (ImageJ v2.16.0; RRID:SCR_003070), expressed as fold-change relative to the **0 µM** condition; bars show mean with individual data points overlaid. Statistical annotations: **ns**, not significant; ****** *p* < 0.01.

**
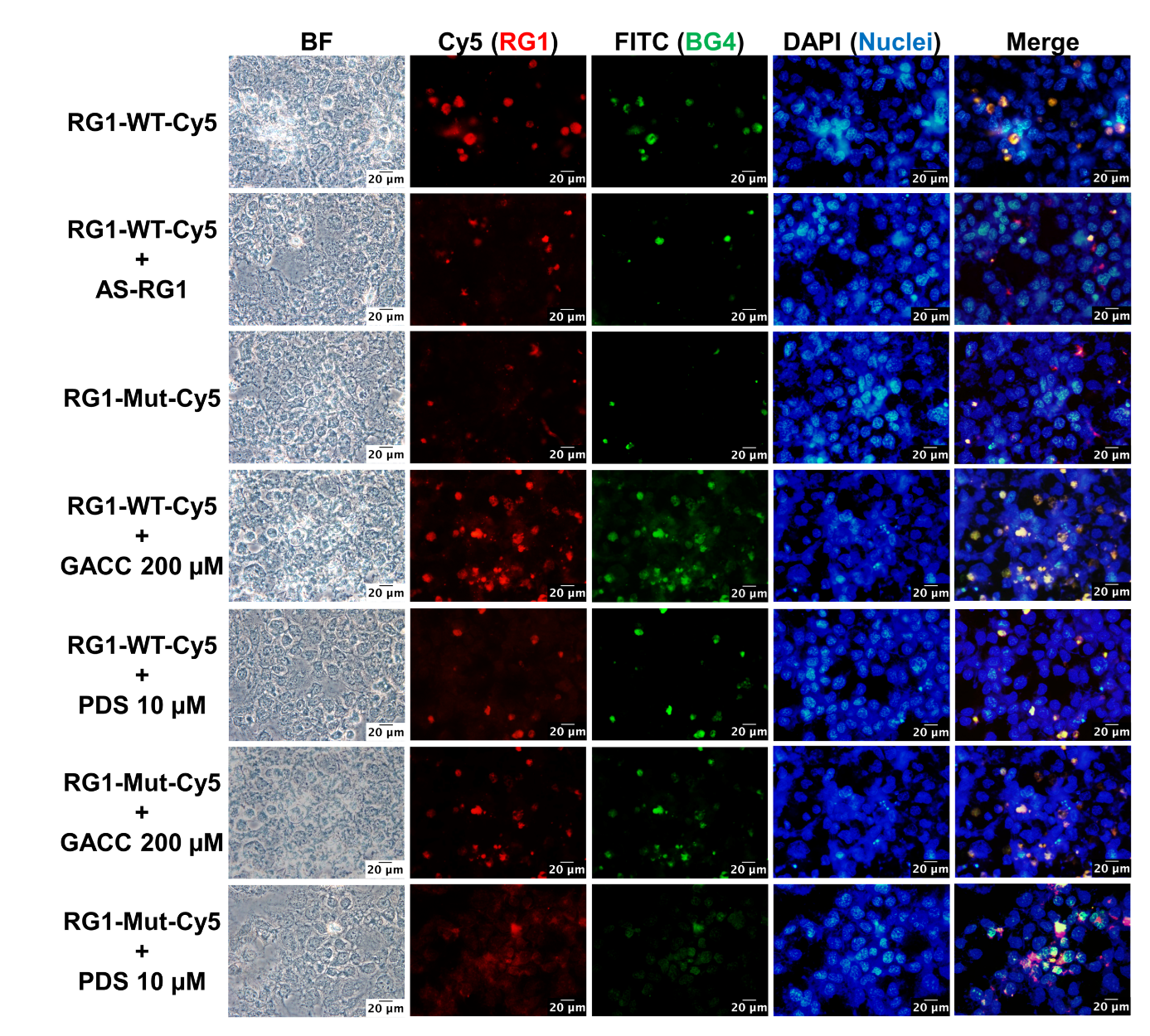
Figure S2. BG4 immunofluorescence indicates increased nuclear G4 signal in Hep3B cells following GACC treatment.** Hep3B cells were transfected with **Cy5-labeled RG-1 G4 oligonucleotides** and treated with **GACC (200 µM)** for **24 h**. Cells were immunostained with **BG4 (anti-G4)** and counterstained with **DAPI**. Representative images were acquired under identical microscope and exposure settings across conditions. Scale bar, **20 µm**.

**
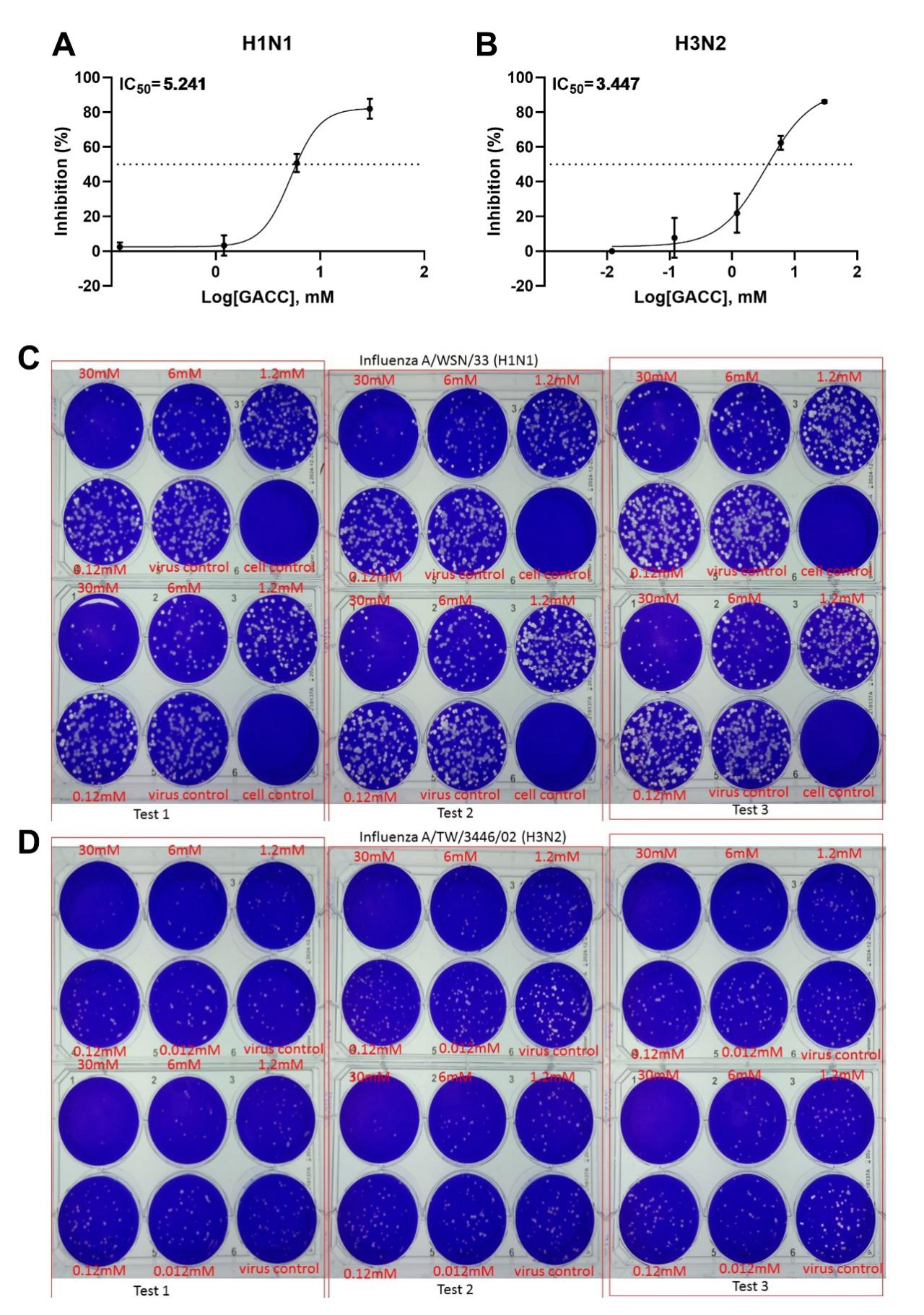
Figure S3. Exploratory antiviral activity of GACC against influenza A viruses *in vitro*. (A–B)** Dose–response curves for GACC against influenza A H1N1 and H3N2 (strains indicated in Methods) measured by plaque-based readouts. **(C–D)** Quantification of viral titers following GACC treatment for H1N1 **(C)** and H3N2 **(D)**. Data are presented as mean ± SEM from the indicated number of independent experiments. IC₅₀ values were derived by nonlinear regression as described in Methods. These results are preliminary and are provided as exploratory findings.

# Table S1. Resources & RRIDs

| **Resource** | **Supplier/Source** | **Catalog/Model** | **RRID** | **Location** |
| --- | --- | --- | --- | --- |
| Anti-DNA G-quadruplex structures, clone BG4 | Merck Millipore | MABE917 |  | Burlington, MA, USA |
| Lipofectamine 3000 | Invitrogen | L3000015 |  | Waltham, MA, USA |
| HepG2 cell line | BCRC (FIRDI) | 60025 | RRID:CVCL_0027 | Hsinchu City, Taiwan |
| PLC/PRF/5 cell line | BCRC (FIRDI) | 60223 | RRID:CVCL_0485 | Hsinchu City, Taiwan |
| Hep3B2.1-7 (Hep3B) cell line | BCRC (FIRDI) | 60434 | RRID:CVCL_0326 | Hsinchu City, Taiwan |
| THLE-2 cell line | ATCC | CRL-2706 | RRID:CVCL_3803 | **Manassas, VA, USA** |
| MDCK (NBL-2) cell line | ATCC | CCL-34 | RRID:CVCL_0422 | **Manassas, VA, USA** |
| BG4 scFv (FLAG-tagged) | Absolute Antibody | Ab00174-1.1 | — | Oxford, United Kingdom |
| Anti-DYKDDDDK (FLAG) (Rabbit mAb) | Cell Signaling Technology | #2368 | — | Danvers, MA, USA. |
| Goat Anti-Rabbit IgG (H+L), DyLight 488 | GeneTex | GTX213110-04 | — | Hsinchu, Taiwan |
| DAPI | Invitrogen | D1306 | — | Waltham, MA, USA |
| NucleoSpin® RNA Midi kit | MACHEREY-NAGEL | 740962 | — | Düren, Germany |
| rDNase Set | MACHEREY-NAGEL | 740963 | — | Düren, Germany |
| iScript™ cDNA Synthesis Kit | Bio-Rad | 1708891 | — | Hercules, CA, USA |
| Fast SYBR™ Green Master Mix | Applied Biosystems | 4385612 | — | Waltham, MA, USA |
| TRIzol™ Reagent | Invitrogen | 15596026 | — | Waltham, MA, USA |
| Taq DNA polymerase, recombinant | Invitrogen | 11615-010 | — | Waltham, MA, USA |
| Metformin hydrochloride | MedChemExpress (MCE) | HY-17471A/CS-1851 | — | Monmouth Junction, NJ, USA. |
| Regorafenib | TargetMol | T1792 | — | Wellesley Hills/Boston, MA, USA |
| Pyridostatin (RR82) trifluoroacetate salt (PDS) | Selleck Chemicals | S7444 | — | Houston, TX, USA |
| Glutamic acid–chelated cobalt (GACC) | Amelio Biomedical | — | — | New Taipei City, Taiwan |
| Vybrant™ DiI Cell-Labeling Solution | Invitrogen | V22885 | — | Waltham, MA, USA |
| DMEM, high glucose | Gibco (Thermo Fisher Scientific) | 11965-092 | — | Waltham, MA, USA |
| Fetal Bovine Serum (FBS), qualified | Gibco (Thermo Fisher Scientific) | 16000-044 | — | Waltham, MA, USA |
| Penicillin–Streptomycin (100×) | Gibco (Thermo Fisher Scientific) | 15140-122 | — | Waltham, MA, USA |
| Fiji (ImageJ-based distribution) | National Institutes of Health (NIH) | v2.16.0 | RRID:SCR_003070 | Bethesda, MD, USA |
| GraphPad Prism | Dotmatics/GraphPad | 10.3.1 | RRID:SCR_002798 | San Diego, CA, USA |
| RCSB Protein Data Bank | RCSB | — | RRID:SCR_012820 | Piscataway, NJ, USA |
| *Tg(fabp10a:EGFP-mCherry) nn1000* | ZFIN | — | RRID:ZDB-TGCONSTRUCT-131205-1 | University of Oregon, Eugene, OR, USA |
| HSPR transgenic zebrafish | NHRI internal resource | — | — | Zhunan, Miaoli County, Taiwan |
| Applied Biosystems™ ViiA™ 7 Real-Time PCR System | Thermo Fisher Scientific | — | — | Waltham, MA, USA |
| Bruker D8 Venture diffractometer | Bruker AXS | — | — | Karlsruhe, Germany |
| Taiwan Zebrafish Core Facility at NTHU-NHRI | NHRI internal resource | — | — | Zhunan, Miaoli County, Taiwan |
| Instrumentation Center, National Taiwan University | National Taiwan University | — | — | Taipei, Taiwan |
| SGS Taiwan | — | — | — | Taipei, Taiwan |
| Development Center for Biotechnology (DCB), Taiwan | — | — | — | Taipei, Taiwan |
| AminoMatrix Labs | — | — | — | New Taipei City, Taiwan |
| Cy5-labeled KRAS-G4 oligonucleotides | MDBio, Inc. | — | — | Taipei, Taiwan |
| Microscope/Camera for zebrafish imaging | Leica DMIRB inverted fluorescence microscope / Olympus cooled digital color camera DP73 | Leica DMIRB; Olympus DP73 | — | Wetzlar, Germany |
| MetaMorph | Molecular Devices | 7.10.1.161 | RRID:SCR_002368 | San Jose, CA, USA |

**Note:**

1. RRID: Research Resource Identifier.

2. ‘—’ indicates not available or not applicable.

3. Software versions are provided in the Catalog/Model column where relevant.

4. Fiji is an ImageJ-based distribution; version shown above refers to the Fiji/ImageJ build used.

**Table S2. Crystal data and experimental details for GACC.**

| **Parameter** | | **Value** |
| --- | --- | --- |
| Empirical formula | C_5_ H_11_ Co N O_6_ | |
| Formula weight | 240.08 | |
| Crystal system | Orthorhombic | |
| Space group | P212121 | |
| Unit cell dimensions | a = 7.12700(10) Å | |
|  | b = 10.4397(3) Å | |
|  | c = 11.2621(3) Å | |
| Volume | 837.94(3) Å3 | |
| Z | 4 | |
| F(000) | 492 | |
| Density (calculated) | 1.903 g/cm3 | |
| Wavelength | 0.71073 Å | |
| Cell parameters reflections used | 6952 | |
| Theta range for Cell parameters | 2.86 to 28.31°. | |
| Absorption coefficient | 2.047 /mm | |
| Temperature | 200(2) K | |
| Crystal size | 0.370 × 0.165 × 0.101 mm^3^ | |
|  |  | |
| **Data collection** | | |
| Diffractometer | Bruker D8 Venture | |
| Absorption correction | Semi-empirical from equivalents | |
| Max. and min. transmission | 0.7457 and 0.5926 | |
| No. of measured reflections | 8384 | |
| No. of independent reflections | 2068 [R(int) = 0.0461] | |
| No. of observed [I>2_igma(I)] | 2040 | |
| Completeness to theta = 25.242° | 99.0 % | |
| Theta range for data collection | 3.383 to 28.307° | |
|  |  | |
| **Refinement** | | |
| Final R indices [I>2sigma(I)] | R1 = 0.0276, wR2 = 0.0758 | |
| R indices (all data) | R1 = 0.0279, wR2 = 0.0759 | |
| Goodness-of-fit on F2 | 1.241 | |
| No. of reflections | 2068 | |
| No. of parameters | 163 | |
| No. of restraints | 4 | |
| Absolute structure parameter | 0.10(3) | |
| Largest diff. peak and hole | 0.359 and -0.347 e.Å-3 | |

Note: Values in parentheses are standard uncertainties (s.u.) in the last significant digits.

R1 = Σ||Fo| − |Fc|| / Σ|Fo|; wR2 = [Σw(Fo² − Fc²)² / Σw(Fo²)²]¹ᐟ².

**Table S3. Atomic coordinates ( × 10^4^) and equivalent isotropic displacement parameters (Å^2^ × 10^-3^) for GACC.**

| **Atom label** | **x** | **y** | **z** | **U(eq)** |
| --- | --- | --- | --- | --- |
| Co(1) | 3930(1) | 5640(1) | 4582(1) | 15(1) |
| O(1) | 5276(4) | 7367(3) | 4818(2) | 17(1) |
| O(2W) | 9607(4) | 3517(3) | 5736(2) | 20(1) |
| O(3) | 8131(4) | 5346(3) | 9354(2) | 21(1) |
| O(2) | 6388(4) | 8743(3) | 6153(2) | 18(1) |
| O(4) | 9914(4) | 4655(3) | 7899(2) | 21(1) |
| O(1W) | 3050(4) | 3807(3) | 4780(3) | 25(1) |
| N(1) | 3129(4) | 6103(3) | 6359(3) | 15(1) |
| C(2) | 5397(5) | 7810(3) | 5849(3) | 14(1) |
| C(5) | 8429(5) | 5229(4) | 8265(3) | 17(1) |
| C(1) | 4294(5) | 7144(4) | 6850(3) | 13(1) |
| C(3) | 5642(5) | 6666(4) | 7803(3) | 16(1) |
| C(4) | 7117(5) | 5756(4) | 7325(3) | 20(1) |

**Notes:**

1. Atomic coordinates are given in units of ×10⁴.
2. U(eq) denotes the equivalent isotropic displacement parameter, defined as one third of the trace of the orthogonalized U_ij_ tensor, in units of Å² × 10⁻³.
3. Values in parentheses are estimated standard deviations (e.s.d.’s) in the last significant digits.

**Table S4. Selected bond lengths [Å] and bond angles [°] for GACC.**

| **Bond / Angle** | **Value** |
| --- | --- |
| Co(1)-O(1W) | 2.026(3) |
| Co(1)-O(1) | 2.060(3) |
| Co(1)-O(4)#1 | 2.090(3) |
| Co(1)-O(2)#2 | 2.094(3) |
| Co(1)-N(1) | 2.137(3) |
| Co(1)-O(3)#1 | 2.348(3) |
| O(1)-C(2) | 1.253(5) |
| O(3)-C(5) | 1.250(5) |
| O(2)-C(2) | 1.251(4) |
| O(4)-C(5) | 1.285(4) |
| N(1)-C(1) | 1.475(5) |
| C(2)-C(1) | 1.540(5) |
| C(5)-C(4) | 1.516(5) |
| C(1)-C(3) | 1.525(5) |
| C(3)-C(4) | 1.516(5) |
| O(1W)-Co(1)-O(1) | 163.11(12) |
| O(1W)-Co(1)-O(4)#1 | 94.75(12) |
| O(1)-Co(1)-O(4)#1 | 93.57(11) |
| O(1W)-Co(1)-O(2)#2 | 93.80(12) |
| O(1)-Co(1)-O(2)#2 | 100.63(11) |
| O(4)#1-Co(1)-O(2)#2 | 91.60(10) |
| O(1W)-Co(1)-N(1) | 91.61(12) |
| O(1)-Co(1)-N(1) | 78.78(11) |
| O(4)#1-Co(1)-N(1) | 171.25(12) |
| O(2)#2-Co(1)-N(1) | 93.97(11) |
| O(1W)-Co(1)-O(3)#1 | 82.78(11) |
| O(1)-Co(1)-O(3)#1 | 88.99(11) |
| O(4)#1-Co(1)-O(3)#1 | 58.96(10) |
| O(2)#2-Co(1)-O(3)#1 | 149.73(10) |
| N(1)-Co(1)-O(3)#1 | 116.13(11) |
| C(2)-O(1)-Co(1) | 118.4(2) |
| C(5)-O(3)-Co(1)#3 | 85.0(2) |
| C(2)-O(2)-Co(1)#4 | 128.3(2) |
| C(5)-O(4)-Co(1)#3 | 95.9(2) |
| C(1)-N(1)-Co(1) | 111.5(2) |
| O(2)-C(2)-O(1) | 125.5(3) |
| O(2)-C(2)-C(1) | 116.1(3) |
| O(1)-C(2)-C(1) | 118.4(3) |
| O(3)-C(5)-O(4) | 120.0(3) |
| O(3)-C(5)-C(4) | 123.0(3) |
| O(4)-C(5)-C(4) | 117.0(3) |
| N(1)-C(1)-C(3) | 112.2(3) |
| N(1)-C(1)-C(2) | 110.2(3) |
| C(3)-C(1)-C(2) | 110.0(3) |
| C(4)-C(3)-C(1) | 113.1(3) |
| C(5)-C(4)-C(3) | 114.0(3) |
| O(4)-C(5)-C(4) | 117.0(3) |

**Notes:**
Bond lengths are given in ångströms (Å) and bond angles in degrees (°).
Values in parentheses are estimated standard deviations (e.s.d.’s) in the last significant digits.
Symmetry transformations used to generate equivalent atoms are listed above.

**Table S5. Anisotropic displacement parameters (Å^2^× 10^-^**³**) for GACC.**

| **Atom label** | **U11** | **U22** | **U33** | **U23** | **U13** | **U12** |
| --- | --- | --- | --- | --- | --- | --- |
| Co(1) | 14(1) | 16(1) | 14(1) | 0(1) | 1(1) | 0(1) |
| O(1) | 20(1) | 17(1) | 15(1) | 1(1) | 2(1) | -6(1) |
| O(2W) | 21(1) | 21(1) | 18(1) | -4(1) | -1(1) | 2(1) |
| O(3) | 22(1) | 28(2) | 15(1) | 1(1) | -2(1) | 6(1) |
| O(2) | 18(1) | 18(1) | 20(1) | -2(1) | 2(1) | -6(1) |
| O(4) | 19(1) | 30(2) | 15(1) | -1(1) | -4(1) | 12(1) |
| O(1W) | 20(1) | 19(1) | 38(2) | 4(1) | 7(1) | 2(1) |
| N(1) | 13(1) | 18(1) | 15(1) | 2(1) | 1(1) | -3(1) |
| C(2) | 9(1) | 13(2) | 18(2) | 4(1) | 0(1) | 1(1) |
| C(5) | 16(2) | 18(2) | 16(2) | 1(1) | -2(1) | 2(1) |
| C(1) | 12(2) | 16(2) | 12(2) | 1(1) | 0(1) | 1(1) |
| C(3) | 16(2) | 20(2) | 12(2) | 0(1) | -1(1) | 3(1) |
| C(4) | 20(2) | 26(2) | 14(2) | 2(2) | -2(1) | 9(2) |

**Notes:**

1. Anisotropic displacement parameters Uij are given in units of Å² × 10^-^³.
2. The anisotropic displacement factor exponent takes the form: −2π²[h²a²U11 + k²b²U22 + l²c²U33 + 2hk abU12 + 2hl acU13 + 2kl bcU23].*
3. Values in parentheses are estimated standard deviations (e.s.d.’s) in the last significant digits.

**Table S6. Hydrogen coordinates ( × 10^4^) and isotropic displacement parameters (Å^2^× 10^-^**³**) for GACC.**

| **Hydrogen label** | **x** | **y** | **z** | **U(eq)** |
| --- | --- | --- | --- | --- |
| H(1) | 3010(70) | 5500(60) | 6970(50) | 26(13) |
| H(2) | 1960(70) | 6320(50) | 6310(40) | 15(11) |
| H(3) | 3460(70) | 7700(50) | 7110(50) | 21(12) |
| H(4) | 5080(80) | 6220(60) | 8380(50) | 27(13) |
| H(5) | 6260(70) | 7390(40) | 8270(40) | 17(11) |
| H(6) | 6510(60) | 5010(50) | 6890(40) | 13(11) |
| H(7) | 7800(80) | 6110(60) | 6690(50) | 35(15) |
| H(1W) | 1880(50) | 3590(60) | 5110(50) | 36(15) |
| H(2W) | 3800(110) | 3090(60) | 4510(80) | 100(30) |
| H(3W) | 8760(70) | 3960(60) | 5210(50) | 47(17) |
| H(4W) | 9550(110) | 3900(70) | 6530(40) | 70(20) |

**Notes:**
1. Hydrogen coordinates are given in units of ×10⁴.

2. U(eq) denotes the equivalent isotropic displacement parameter, defined as one third of the trace of the orthogonalized U_ij_ tensor, in units of Å² × 10^-^³.

3. Values in parentheses are estimated standard deviations (e.s.d.’s) in the last significant digits.

**Table S7. IC₅₀ Values of GACC in Various Human Cancer Cell Lines Demonstrating Broad-Spectrum Anticancer Activity**

| **Cancer** | **Cell Line** | **IC_50_** |
| --- | --- | --- |
| Human Glioblastoma | U87 | 42 µM |
| Lung Cancer | H226 | 44.5 µM |
| Chronic Myelogenous Leukemia | K562 | 54.5 µM |
| Cervical Cancer | HeLa | 73.7 µM |
| Hypopharyngeal Carcinoma | FaDu | 74.6 µM |
| Human Malignant Melanoma | A375 | 102 µM |

**Note:** IC_50_, half-maximal inhibitory concentration.

**Table S8. Exploratory antibacterial and antifungal activity of GACC against selected WHO-priority pathogens (SGS Taiwan).**

| **Organism** | **Original inoculum (CFU/mL)** | **Contact time** | **Counts at contact time (CFU/mL)** | **Antimicrobial activity R (%)** |
| --- | --- | --- | --- | --- |
| *Staphylococcus aureus* (MRSA) | 8.5 × 10^5^ | 8 h | 7.7 × 10^5^ | 9.4 |
|  |  | 24 h | 1.8 × 10^4^ | 97.9 |
|  |  | 48 h | 9.0 × 10^2^ | 99.9 |
| *Candida albicans* | 1.3 × 10^5^ | 8 h | 1.3 × 10^5^ | <1 |
|  |  | 24 h | 4.4 × 10^4^ | 66.2 |
|  |  | 48 h | 9.5 × 10^3^ | 92.7 |
| *Candida auris* | 6.6 × 10^5^ | 8 h | 4.2 × 10^5^ | 36.4 |
|  |  | 24 h | 2.6 × 10^5^ | 60.6 |
|  |  | 48 h | 7.8 × 10^4^ | 88.2 |
| *Pseudomonas aeruginosa* | 3.6 × 10^5^ | 8 h | 1.3 × 10^3^ | 99.6 |
|  |  | 24 h | <1 | >99.9 |
|  |  | 48 h | <1 | >99.9 |
| *Klebsiella pneumoniae* | 4.4 × 10^5^ | 8 h | 3.1 × 10^5^ | 29.5 |
|  |  | 24 h | 8.5 × 10^4^ | 80.7 |
|  |  | 48 h | 9.9 × 10^3^ | 97.8 |
| *Escherichia coli* | 7.0 × 10^5^ | 8 h | 4.8 × 10^5^ | 31.4 |
|  |  | 24 h | 5.9 × 10^4^ | 91.6 |
|  |  | 48 h | 1.4 × 10^4^ | 98.0 |

**Note:** Antimicrobial susceptibility testing was performed by SGS Taiwan using standardized broth microdilution assays against methicillin-resistant Staphylococcus aureus (MRSA), Pseudomonas aeruginosa, Klebsiella pneumoniae, Escherichia coli, Candida albicans, and Candida auris. The figure shows colony-forming unit (CFU) counts and calculated inhibition rates (R%) at the indicated time points. Under the tested conditions, GACC produced high levels of growth inhibition at 48 h for MRSA, *P. aeruginosa*, *K. pneumoniae*, and *E. coli* (≥97%), and partial inhibition for *C. albicans* (92.7%) and *C. auris* (88.2%). These data indicate antimicrobial activity *in vitro*; mechanistic basis, selectivity, and therapeutic relevance were not evaluated in this study and warrant further investigation.
